# Role of Plasmodium falciparum Kelch 13 Protein Mutations in P. falciparum Populations from Northeastern Myanmar in Mediating Artemisinin Resistance

**DOI:** 10.1128/mBio.01134-19

**Published:** 2020-02-25

**Authors:** Faiza Amber Siddiqui, Rachasak Boonhok, Mynthia Cabrera, Huguette Gaelle Ngassa Mbenda, Meilian Wang, Hui Min, Xiaoying Liang, Junling Qin, Xiaotong Zhu, Jun Miao, Yaming Cao, Liwang Cui

**Affiliations:** a Department of Internal Medicine, University of South Florida, Tampa, Florida, USA; b Department of Biochemistry & Molecular Biology, The Pennsylvania State University, University Park, Pennsylvania, USA; c College of Basic Medical Sciences, China Medical University, Shenyang, Liaoning, China; NIAID/NIH

**Keywords:** PfK13, *Plasmodium falciparum*, artemisinin resistance, China-Myanmar border, mutations, drug resistance

## Abstract

Mutations in the Plasmodium falciparum Kelch 13 (PfK13) protein are associated with artemisinin resistance. PfK13 is essential for asexual erythrocytic development, but its function is not known. We tagged the PfK13 protein with green fluorescent protein in P. falciparum to study its expression and localization in asexual and sexual stages. We used a new antibody against PfK13 to show that the PfK13 protein is expressed ubiquitously in both asexual erythrocytic stages and gametocytes and is localized in punctate structures, partially overlapping an endoplasmic reticulum marker. We introduced into the 3D7 strain four PfK13 mutations (F446I, N458Y, C469Y, and F495L) identified in parasites from the China-Myanmar border area and characterized the *in vitro* artemisinin response phenotypes of the mutants. We found that all the parasites with the introduced PfK13 mutations showed higher survival rates in the ring-stage survival assay (RSA) than the wild-type (WT) control, but only parasites with N458Y displayed a significantly higher RSA value (26.3%) than the WT control. After these PfK13 mutations were reverted back to the WT in field parasite isolates, all revertant parasites except those with the C469Y mutation showed significantly lower RSA values than their respective parental isolates. Although the 3D7 parasites with introduced F446I, the predominant PfK13 mutation in northern Myanmar, did not show significantly higher RSA values than the WT, they had prolonged ring-stage development and showed very little fitness cost in *in vitro* culture competition assays. In comparison, parasites with the N458Y mutations also had a prolonged ring stage and showed upregulated resistance pathways in response to artemisinin, but this mutation produced a significant fitness cost, potentially leading to their lower prevalence in the Greater Mekong subregion.

## Abstract (precis)

Artemisinin resistance has emerged in Southeast Asia, endangering the substantial progress in malaria elimination worldwide. It is associated with mutations in the PfK13 protein, but how PfK13 mediates artemisinin resistance is not completely understood. Here we used a new antibody against PfK13 to show that the PfK13 protein is expressed in all stages of the asexual intraerythrocytic cycle as well as in gametocytes and is partially localized in the endoplasmic reticulum. By introducing four PfK13 mutations into the 3D7 strain and reverting these mutations in field parasite isolates, we determined the impacts of these mutations identified in the parasite populations from northern Myanmar on the ring stage using the *in vitro* ring survival assay. The introduction of the N458Y mutation into the 3D7 background significantly increased the survival rates of the ring-stage parasites but at the cost of the reduced fitness of the parasites. Introduction of the F446I mutation, the most prevalent PfK13 mutation in northern Myanmar, did not result in a significant increase in ring-stage survival after exposure to dihydroartemisinin (DHA), but these parasites showed extended ring-stage development. Further, parasites with the F446I mutation showed only a marginal loss of fitness, partially explaining its high frequency in northern Myanmar. Conversely, reverting all these mutations, except for the C469Y mutation, back to their respective wild types reduced the ring-stage survival of these isolates in response to *in vitro* DHA treatment.

## INTRODUCTION

The emergence and spread of artemisinin (ART)-resistant Plasmodium falciparum parasites in the Greater Mekong subregion (GMS) of Southeast Asia threaten the substantial progress made toward malaria elimination (1). Reports of ART resistance first surfaced from northwest Cambodia in about 2006 (2, 3), which was followed by a series of reports of ART-resistant parasites appearing in other parts of the GMS (4–10). Although resistance is not yet detected in Africa, cross-sectional surveys in Uganda detected P. falciparum parasites with elevated *ex vivo* ring-stage survival rates (11), prompting further follow-up studies in Africa. Since ART-based combination therapies (ACTs) are the frontline treatment for falciparum malaria, close surveillance of ART resistance is warranted. It is therefore a priority to track the evolution and spread of ART resistance so that strategies can be designed to prevent the further spread of the resistant parasites.

According to WHO guidelines, artemisinin resistance is defined as delayed parasite clearance following treatment with an artesunate monotherapy or with an artemisinin-based combination therapy (2, 12). ART-resistant parasites have a parasite clearance half-life of >5 h after ART treatment, whereas that for sensitive parasites is ∼2 h. As a result, patients infected with ART-resistant parasites often remain parasite positive 3 days after treatment (13). Variations in host immunity also influence parasite clearance after ART treatment and the interpretation of emerging ART resistance (14–18). The delayed-clearance phenotype cannot be captured by the traditional *in vitro/ex vivo* drug assays that measure parasite proliferation but is associated with the results of the ring-stage survival assay (RSA), which determines the percentage of early (0 to 3 h postinvasion) ring-stage parasites (RSA_0–3_ _h_) able to survive a single 6-h pulse of 700 nM dihydroartemisinin (DHA), the active metabolite of ART (19). Whereas genome-wide association studies, gene manipulation experiments, and studies of field isolates with resistant phenotypes have led to the identification of a number of genetic loci and proteins (e.g., ATG18, coronin, pfap2μ, falcipain 2a) predicted to play a role in ART resistance (10, 20–28), now the P. falciparum Kelch 13 (PfK13) protein appears to be a major player, as mutations in its propeller domain have been associated with ART resistance (29). Some of the PfK13 mutations, including Y493H, R539T, I543T, and C580Y, that were associated with clinical ART resistance have been genetically confirmed to confer elevated RSA_0–3_ _h_ survival rates *in vitro* (30, 31). ART drugs are known to induce oxidative stress and cellular damage in P. falciparum, but how the mutated forms of the PfK13 protein allow the parasites to tolerate this oxidative assault is not yet clear. PfK13 has been shown to be essential for parasite survival (32, 33). Conditional knockout of PfK13 leads to rapid growth arrest at the ring stage, with parasites gradually turning into condensed forms, suggesting that PfK13 is required for the survival of the ring stage and/or for the ring stage-to-trophozoite transition in P. falciparum (32). Interestingly, PfK13 mutant parasites can reprogram their intraerythrocytic development cycle (IDC) to have a prolonged ring stage (34–36). Different mechanisms of ART resistance, which may act cooperatively, have been proposed, including the upregulation of the unfolded protein response pathway (37), decreased expression of genes involved in DNA replication (31), and an enhanced stress response, comprising the ubiquitin/proteasome pathway (34). Phosphorylation of the parasite α subunit of eukaryotic initiation factor 2 (eIF2α) and the interaction of phosphatidylinositol 3-kinase (PI3K) with PfK13 have also been proposed to play a role in ART resistance (38–40). Thus, it appears that PfK13 is central to multiple intracellular processes in P. falciparum.

More than 200 PfK13 mutations have been reported across the globe (7, 8, 41–43), but only some of these mutations (especially those prevalent in the GMS) have been associated with slow parasite clearance and reduced ART susceptibility in RSA. Even within geographical proximity of the GMS, PfK13 mutations are highly diverse and region specific, probably reflecting the different drug use histories and the divergent evolutionary trajectories of these parasites. PfK13 C580Y is the predominant mutation in parasites from the Thai-Cambodian border area, southern Laos, and Vietnam (7, 29, 44, 45), whereas the Y493H, R539T, and I543T mutations are more common in the Thai-Cambodian/Thai-Myanmar border areas (8, 9, 29, 46). The F446I mutation is most prevalent in northern Myanmar and the China-Myanmar border area, and some reports showed its association with ART resistance (43, 47, 48). In our earlier study, several mutations present sporadically in the China-Myanmar border area (e.g., N458Y, C469Y, and F495L) showed relatively high RSA values (43). The N458Y mutation was also identified in Thailand and Cambodia and showed a significant association with prolonged parasite clearance half-lives in clinical studies and elevated *in vitro* RSA values (42, 43, 49, 50). We identified one field isolate from the China-Myanmar border area with the C469Y mutation showing high *in vitro* RSA values (43, 51). It is noteworthy that this mutation was also reported recently from Uganda (11, 52–54). The F495L mutation was also lately reported from sub-Saharan Africa (47, 52, 53, 55, 56), but it was not associated with ART resistance. In the China-Myanmar border area, the F446I, N458Y, and C469Y mutations have all been associated with positive parasitemia on day 3 after treatment with an ACT (47). Here we aimed to determine whether these PfK13 mutations are associated with *in vitro* ART susceptibility by introducing them into laboratory strain 3D7 and reverting these mutations to the wild type (WT) in respective field isolates.

## RESULTS

### Expression and localization of PfK13.

**FIG 1:**
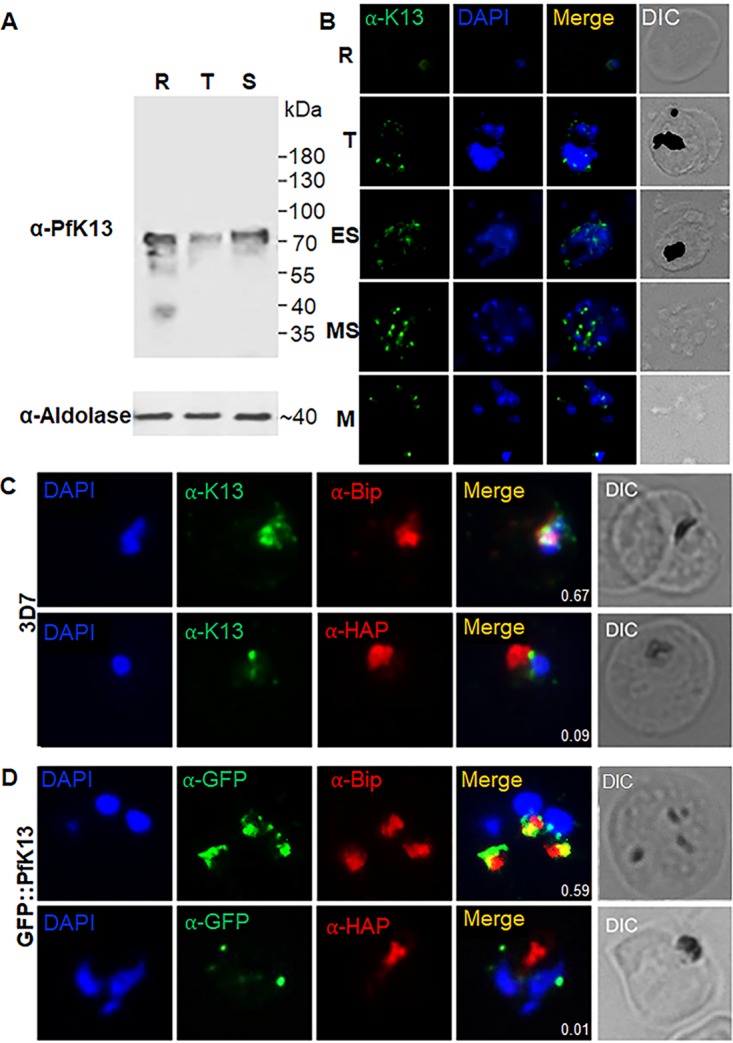
Expression and localization of PfK13 during asexual erythrocytic growth. (A) Western blots of PfK13 expression at the ring (R), trophozoite (T), and schizont (S) stages of 3D7 parasites with anti-PfK13 antibodies. Aldolase was used as the control for equal protein loading from different stages. (B) Localization of PfK13 in the ring (R), trophozoite (T), early schizont (ES), mature schizont (MS), and merozoite (M) stages by IFA with anti-PfK13 antibodies. (C) Colocalization analysis of PfK13 with BiP (an ER marker) and HAP (a food vacuole marker) in 3D7 using anti-PfK13 antibodies. (D) Colocalization analysis of PfK13 with BiP and HAP in GFP::PfK13 parasites using anti-GFP antibodies. DIC, differential interference contrast.

To study the expression and localization of PfK13, we generated affinity-purified rabbit polyclonal antisera against the PfK13 peptide from amino acids (aa) 239 to 257 and a parasite line (GFP::PfK13) in the 3D7 strain with the N terminus of the endogenous PfK13 protein tagged with green fluorescent protein (GFP). To study PfK13 expression, Western blotting was performed with 100 μg of lysates from each asexual stage, including rings, trophozoites, and schizonts, of the 3D7 parasites using anti-PfK13 peptide antibodies. A specific band of ∼83 kDa was detected in the trophozoites and schizonts, corresponding to the predicted size of the endogenous PfK13 protein (Fig. 1A). In ring-stage parasites, however, in addition to the ∼83-kDa major protein band, the anti-PfK13 peptide antibodies also detected three smaller, less abundant protein bands, which may have been degraded or/and processed PfK13 or cross-reacting proteins expressed only in the ring-stage parasites. Compared with the expression of the aldolase protein as the protein loading control, PfK13 protein expression appeared to be relatively consistent throughout the IDC. For validation of the PfK13 expression results detected with the anti-PfK13 peptide antibodies, we tagged PfK13 with GFP at its N terminus using the selection-linked integration (SLI) approach (see Fig. S1A in the supplemental material), as PfK13 is refractory to GFP tagging at the C terminus (32). Using mixed asexual-stage parasites, we confirmed the successful integration of GFP by integration-specific PCR (Fig. S1B). In a Western blot, the anti-GFP antibody detected a major protein band of ∼165 kDa, which agreed with the predicted size of PfK13 fused with two FK506-binding protein (FKBP) domains and GFP, as well as some protein bands of lower molecular weights, suggestive of degraded protein products (Fig. S1C). Western blotting with protein lysates from synchronized rings, trophozoites, schizonts, and purified merozoites of the GFP::PfK13 parasite line using the anti-GFP antibodies consistently detected the presence of the 165-kDa band in all asexual stages (Fig. S2A). Although lower-molecular-weight protein products were detected in all stages, bands that were exclusive to the ring stage were not detected in the GFP::PfK13 parasites.

10.1128/mBio.01134-19.1 FIG S1 (A) Schematic showing the replacement of endogenous PfK13 with N-terminal GFP/PTP-tagged full-length recoded PfK13 (WT or mutated) using the selection-linked integration (SLI) method. Genomic integration of the plasmid results in the expression of a second resistance marker (yeast dihydroorotate dehydrogenase [yDHODH]) that is flanked by two skip peptides (2A), generating separate proteins. Arrows labeled 1 to 5 indicate the primers used for integration-specific PCR. Arrows 6 and 7 indicate the *hsp86* and the *PfK13* endogenous promoters, respectively. hDHFR, human dihydrofolate reductase; FKBP, FK506-binding protein; GFP, green fluorescent protein; PTP, protein C-tobacco etch virus-protein A. (B) PCR performed using genomic DNA from different parasite lines and the primers indicated in panel A to show integration of the plasmid into the correct endogenous locus with primers 1 and 2 (top) and the intact endogenous locus with primers 1 and 4 (bottom). (C) Western blots detecting the full-length PfK13 protein (165 kDa) with an N-terminal 2×FKBP-GFP-2×FKBP tag (left) and 2×FKBP-PTP-2×FKBP tag (right) using anti-GFP and anti-protein C (1:1,000; catalog number A00637; GenScript) antibodies, respectively. (D) Schematic showing the replacement of endogenous PfK13 with N-terminal GFP/PTP-tagged full-length recoded WT PfK13 using selection-linked integration. Genomic integration of the plasmid results in the expression of a second resistance marker (yeast dihydroorotate dehydrogenase [yDHODH]) that is flanked by two skip peptides (2A). Arrows labeled 1 to 5 indicate the primers used for performing integration-specific PCR. Arrows 6 and 7 indicate the *hsp86* and the *PfK13* endogenous promoters, respectively. hDHFR, human dihydrofolate reductase; FKBP, FK506-binding protein; GFP, green fluorescent protein; PTP, protein C-tobacco etch virus-protein A. (E) PCR performed using genomic DNA from different parasite lines and the primers indicated in panel A to show integration into the correct endogenous locus with primers 1 and 5 (top) and the intact endogenous locus with primers 1 and 4 (bottom). (F) IFAs detecting the expression of GFP::PfK13 WT in transgenic field isolates using anti-GFP antibodies. Nuclei are stained with DAPI (4′,6-diamidino-2-phenylindole; blue). Download FIG S1, PPT file, 1.0 MB. Copyright © 2020 Siddiqui et al. 2020 Siddiqui et al. This content is distributed under the terms of the Creative Commons Attribution 4.0 International license.

10.1128/mBio.01134-19.2 FIG S2 Expression and localization of GFP::PfK13 during asexual erythrocytic stages. (A) Western blots of PfK13 expression in GFP::PfK13 probed with anti-GFP antibodies. Aldolase was used as the control for equal parasite protein loading. Lysates were prepared from synchronized ring (R), trophozoite (T), schizont (S), and merozoite (M) stages of GFP::PfK13 parasites and mixed asexual stages of the 3D7 parasite. (B) IFA of asexual-stage parasites of the GFP::PfK13 line probed with anti-GFP antibodies. (C). IFA of stage II to V gametocytes of the GFP::PfK13 line probed with anti-GFP antibodies. PfK13 was detected in all asexual stages and gametocytes and was localized in punctate structures in the cytoplasm. The number of puncta increases from the ring to the schizont stage. Download FIG S2, PPT file, 1.2 MB. Copyright © 2020 Siddiqui et al. 2020 Siddiqui et al. This content is distributed under the terms of the Creative Commons Attribution 4.0 International license.

To investigate the subcellular localization of PfK13, an indirect immunofluorescence assay (IFA) and live-cell imaging were performed with both asexual-stage parasites and gametocytes. Probing of the 3D7 parasites with the anti-PfK13 antibodies showed punctate staining in the cytoplasm of the parasites, and the number of puncta increased from the ring stage to the schizont stage (Fig. 1B). Live-cell imaging of the GFP::PfK13 line showed a similar distribution pattern of the GFP fluorescent puncta (Fig. S2B). These puncta did not overlap with the parasite nuclei, suggesting that they were localized in the cytoplasm. Similarly, live-cell imaging of gametocytes in the GFP::PfK13 parasite line identified multiple punctate green fluorescent foci throughout gametocyte development (Fig. S2C). To further define the PfK13 localization, we performed colocalization analysis of PfK13 with an endoplasmic reticulum (ER) marker (heat shock protein 70 [BiP]) and a food vacuole marker (histoaspartic protease [HAP]). The results obtained using anti-PfK13 antibodies in 3D7 parasites showed a partial colocalization of PfK13 with the ER marker (Pearson’s correlation coefficient, 0.575) and an insignificant overlap with the food vacuole marker (Pearson’s correlation coefficient, <0.3) (Fig. 1C). IFA performed in the GFP::PfK13 line further confirmed a partial colocalization with the ER marker (Pearson’s correlation coefficient, 0.524) and much less overlap with the food vacuole marker (Pearson’s correlation coefficient, <0.3) (Fig. 1D).

### Proteins potentially associated with PfK13.

To identify proteins that are associated with PfK13, we performed immunoprecipitation (IP) with protein lysates of asexual blood-stage parasites from the GFP::PfK13 line using GFP-Trap beads followed by liquid chromatography-tandem mass spectrometry (LC-MS/MS) analysis. Using a false discovery rate (FDR) of 1%, 34 proteins that were common in all three biological replicates were identified (Table 1). Among them, 10 proteins are involved in the unfolded protein response pathway. Other proteins are predicted to play roles in transcription, translation, and other biosynthetic pathways. Three proteins, BiP, protein disulfide isomerase (ERp72), and the putative endoplasmin (GRP94), are potentially involved in the *Plasmodium* reactive oxidative stress complex. Interestingly, among these proteins possibly associated with PfK13, 7 and 4 proteins were found in the up- and downregulated gene categories, respectively, in the transcriptome analysis of ART-resistant field isolates (Table 1) (37).

**TABLE 1:** Proteins identified from affinity purification of GFP::PfK13 parasites followed by mass spectrometry^*a*^

Protein name	Identifier	Predicted function
Kelch protein K13	PF3D7_1343700	Unfolded protein response/protein binding
Heat shock protein 70 (BiP)	PF3D7_0917900	Unfolded protein response
DnaJ protein	PF3D7_0629200	Unfolded protein response/protein binding
Protein disulfide isomerase (ERp72)	PF3D7_0827900	Protein folding
40S ribosomal protein S16	PF3D7_0813900	Translation/ structural constituent of ribosome
40S ribosomal protein S19	PF3D7_0422400	Organelle assembly/translation/structural constituent of ribosome
40S ribosomal protein S11	PF3D7_0516200	Organelle assembly/translation/structural constituent of ribosome
Plasmepsin II	PF3D7_1408000	Aspartic protease
14-3-3 protein	PF3D7_0818200	Protein binding
DNA/RNA-binding protein Alba 3	PF3D7_1006200	Transcription factor/protein binding/mRNA binding
*S*-Adenosylmethionine synthase	PF3D7_0922200	Cellular biosynthetic process/methionine adenosyltransferase activity
Parasitophorous vacuolar protein 1	PF3D7_1129100	Food vacuole protein/protein binding
Heat shock protein 70	PF3D7_0818900	Unfolded protein response/ATPase activity
Heat shock protein 70	PF3D7_1134000	Unfolded protein response
Endoplasmin homolog (GRP94)	PF3D7_1222300	Unfolded protein response
Merozoite surface protein 1	PF3D7_0930300	Protein binding/invasion/protein-containing complex binding
High-molecular-weight rhoptry protein 2	PF3D7_0929400	Protein binding/invasion/protein-containing complex binding
T-complex protein 1 subunit gamma	PF3D7_1229500	Protein folding/chaperonin/unfolded protein binding
26S protease regulatory subunit 8	PF3D7_1248900	Transcription preinitiation complex assembly
26S protease regulatory subunit 6B	PF3D7_0413600	Transcription preinitiation complex assembly
Elongation factor 1-alpha	PF3D7_1357000	Translation elongation factor/RNA binding
Elongation factor 2	PF3D7_1451100	Translation elongation factor/RNA binding
Eukaryotic initiation factor 4A	PF3D7_1468700	Translation initiation/mRNA binding
40S ribosomal protein S3a	PF3D7_0322900	Translation/structural constituent of ribosome
40S ribosomal protein S15A	PF3D7_0316800	Translation/structural constituent of ribosome
DNA/RNA-binding protein Alba 1	PF3D7_0814200	Transcription factor/translation regulator activity, nucleic acid binding/mRNA binding
60S acidic ribosomal protein P0	PF3D7_1130200	Organelle assembly
60S ribosomal protein L12	PF3D7_0517000	Organelle assembly/structural molecule activity
Heat shock protein 60	PF3D7_1015600	Unfolded protein response/ATPase activity
Glyceraldehyde-3-phosphate dehydrogenase	PF3D7_1462800	Pyruvate metabolic process/oxidoreductase activity
GTP-binding nuclear protein	PF3D7_1117700	GTPase activity
Protein DJ-1	PF3D7_0627500	Unfolded protein response/protein deglycase activity
Acyl coenzyme A synthetase	PF3D7_0525100	Ligase
l-Lactate dehydrogenase	PF3D7_1324900	Tricarboxylic acid cycle/lactate dehydrogenase activity
60S ribosomal protein L10	PF3D7_1414300	Formation of actively translating ribosomes/structural constituent of ribosome

a Light and dark shading indicate genes up- and downregulated, respectively, in ART-resistant isolates, as reported by Mok et al. (37). All proteins and peptides have an FDR of <1%.

### Introduction of PfK13 mutations and drug sensitivity.

The KARMA consortium identified 108 nonsynonymous mutations in the PfK13 propeller domain in P. falciparum populations worldwide (7). In the GMS, two areas (one including Cambodia, Vietnam, and Laos and the other including western Thailand, Myanmar, and China) have nonoverlapping, region-specific distributions of PfK13 mutations (7, 42, 43). We focused on four PfK13 mutations (F446I, N458Y, C469Y, and F495L) that were prevalent or specific to isolates from northern Myanmar and the China-Myanmar border area (43), and some of these have been associated with delayed parasite clearance (29, 42, 47), day 3 parasitemia, and increased RSA values (43). To test whether these PfK13 mutations are linked to altered ART sensitivity, we introduced these four mutations as well as the C580Y mutation as a positive control into the 3D7 strain using the SLI method together with N-terminal GFP tagging. We also used WT PfK13 as the transfection control. Integration of the different PfK13 versions was confirmed by PCR, Sanger sequencing, and Western blotting (Fig. S1B and S3A).

10.1128/mBio.01134-19.3 FIG S3 Western blot (A), flow cytometry (B), and IFA (C) data showing no significant difference in the level of expression between GFP::WT PfK13 and GFP::mutant PfK13 (F446I, N458Y, C469Y, F495L, and C580Y) in asexual stages. To obtain the mean fluorescence intensities with flow cytometry, 50,000 cells were counted at the trophozoite stage for each parasite line in 2 independent experiments. (D) IFA of GFP::PfK13 in stage III gametocytes of mutant transgenic parasites showing the similar expression and distribution patterns of mutant PfK13. For Western blots and IFA, GFP::PfK13 was detected by anti-GFP antibodies. Download FIG S3, PPT file, 1.8 MB. Copyright © 2020 Siddiqui et al. 2020 Siddiqui et al. This content is distributed under the terms of the Creative Commons Attribution 4.0 International license.

The WT and mutant PfK13 proteins showed expression levels and localization patterns similar to those of the WT recombinant when analyzed by Western blotting, flow cytometry, and live-cell imaging (Fig. S3A to C). The punctate GFP localization patterns of the WT and PfK13 mutant lines also appeared to be similar in gametocyte stages (Fig. S3D). Since PfK13 was reported to concentrate in phosphatidylinositol 3-phosphate (PI3P) vesicles in P. falciparum (40), we wanted to determine whether the PfK13 mutations examined here may affect the distribution of PfK13 and PI3P. Colocalization analysis of GFP::PfK13 with PI3P revealed at least one green fluorescent dot close to the site of PI3P staining, but no overlap of PfK13 staining with PI3P in the WT or the mutant parasite lines was observed at late asexual stages, with Pearson’s correlation coefficient being <0.24 (Fig. S4). The mouse anti-PI3P antibodies used here were unable to detect PI3P at the early ring stage.

10.1128/mBio.01134-19.4 FIG S4 Colocalization analysis of WT and mutated PfK13 with PI3P. Anti-GFP rabbit antibodies and anti-PI3P mouse antibodies were used to probe GFP::PfK13 (green) and PI3P (red) in transgenic 3D7 parasites at the trophozoite (A) and schizont (B) stages. Nuclei are stained with DAPI (4′,6-diamidino-2-phenylindole; blue). Also shown are the bright-ﬁeld (BF) and merged images. Download FIG S4, PPT file, 2.1 MB. Copyright © 2020 Siddiqui et al. 2020 Siddiqui et al. This content is distributed under the terms of the Creative Commons Attribution 4.0 International license.

**FIG 2:**
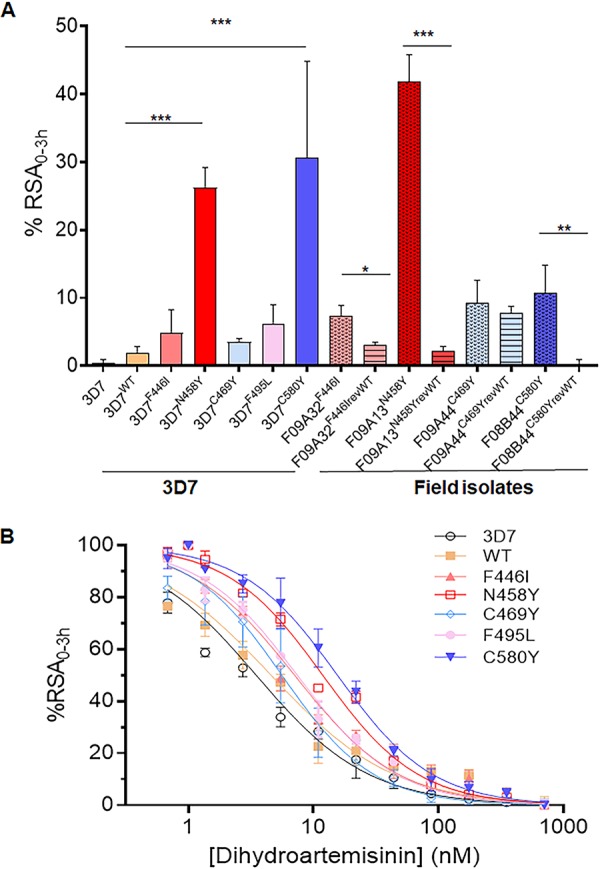
Ring-stage survival assay (RSA) of WT and mutant PfK13 parasites. (A) Survival for 0 to 3 h of early-ring-stage parasites of 3D7, the transfection control with the WT PfK13 gene (3D7^WT^), and 3D7 with one of five mutations (3D7^F446I^, 3D7^N458Y^, 3D7^C469Y^, 3D7^F495L^, 3D7^C580Y^), along with the transgenic field isolates before and after removal of PfK13 mutations (F09A32^F446I^, F09A32^F446IrevWT^, F09A13^N458Y^, F09A13^N458YrevWT^, F09A44^C469Y^, F09A44^C469YrevWT^, F08B44^C580Y^, F08B44^C580YrevWT^), was determined using the standard RSA_0–3_ _h_. The results for each parasite strain or clone were measured in three independent experiments, and the RSA values (in percent) are shown as the mean and standard deviation. Comparison of the results was done using a nonparametric Wilcoxon *t* test, and the *P* values are indicated. *, *P < *0.05; **, *P < *0.01; ***, *P < *0.001. (B) RSA_0–3_ _h_^50%^ of the transgenic 3D7 parasites with WT and mutant PfK13. Parasites were exposed to a 4-h pulse of DHA ranging from 0.6 to 700 nM, and the survival of the parasites under this treatment condition was plotted against the drug concentration. The sigmoid parasite drug response curves were used to estimate the concentration of DHA causing 50% death of the ring-stage parasites (see Table S3 in the supplemental material). Each data point is the mean percent survival from at least three independent experiments performed in duplicate. As a vehicle control, parasites were treated with DMSO.

To determine whether the introduced PfK13 mutations conferred ART resistance in the transfected 3D7 parasite lines, RSA was performed for each parasite line with three biological replicates (Table S2A). Given that the study design introduces two drug selection cassettes into the 3D7 parasites, which could lead to altered drug responses, a transfection control with the WT PfK13 was included for comparison. RSA results showed that the transfection-control parasites with WT PfK13 had ring survival (1.9%) slightly but not significantly higher than that for the parent 3D7 line (0.4%), whereas introduction of PfK13 with the C580Y mutation in the 3D7 line as a positive control conferred an RSA level (30.6%) significantly higher than that for the transfection control (*P < *0.001, nonparametric Wilcoxon matched-pairs test; Fig. 2A). Parasites with F495L, F446I, and C469Y all had RSA values greater than 1% (6.2%, 4.8%, and 3.5%, respectively), albeit they were not statistically significantly higher than those for 3D7 or the WT PfK13 transfection control. Only the N458Y parasites showed a significantly higher ring survival rate (26.3%) than both 3D7 and the transfection control (*P < *0.0001, nonparametric Wilcoxon matched-pairs test; Fig. 2A).

To confirm these findings in isogenic 3D7 parasite lines, we reverted these PfK13 mutations to the WT in the respective field isolates from the China-Myanmar border area (F09A32^F446I^, F09A13^N458Y^, F09A44^C469Y^, F08B44^C580Y^) using the same SLI method (Fig. S1D). These revertant parasites were verified by integration-specific PCR as well as live-cell imaging of GFP fluorescence in asexual stages (Fig. S1E and F). Whereas isolate F09A44^C469Y^ reverted back to the WT (F09A44^C469YrevWT^) showed only a slight, insignificant decrease in the RSA value compared to that for F09A44^C469Y^, all other field strains showed significant decreases when the PfK13 alleles were reverted back to the WT (Fig. 2A). Specifically, RSA values for F09A32^F446I^, F09A13^N458Y^, and F08B44^C580Y^ decreased from 7.3%, 42%, and 10% to 3.1%, 2.2%, and 0.3%, respectively (*P < *0.05) (Fig. 2A).

Since RSA_0–3_ _h_ measures the ring-stage survival only after exposure to 700 nM DHA, we further evaluated the *in vitro* sensitivity of the isogenic 3D7 parasites to a gradient of DHA concentrations ranging from 0.6 to 700 nM for 4 h in the RSA_0–3_ _h_^50%^ (57–59), which determines the amount of DHA required to kill 50% of the parasites. Again, all parasite lines with introduced PfK13 mutations showed increased RSA_0–3_ _h_^50%^ values compared to those for 3D7 (3.5 nM) and the WT PfK13 transfection control (4.6 nM) (Fig. 2B), but only parasites with the C580Y and N458Y mutations had RSA_0–3_ _h_^50%^ values (15.6 and 11.9 nM, respectively; *n* = 3) significantly higher than those for 3D7 or the WT PfK13 transfection control (Table S3).

To determine if altered DHA sensitivity also affected *in vitro* susceptibility to other antimalarial drugs, we determined the 50% inhibitory concentration (IC_50_) values of some commonly used antimalarial drugs for the 3D7 parasite lines carrying the introduced PfK13 mutations using the traditional SYBR green I-based drug sensitivity assay (Table 2). Compared to both 3D7 and the PfK13 transfection-control parasites, some parasites with PfK13 mutations appeared to be slightly more sensitive to chloroquine (CQ) (F495L) and mefloquine (MFQ) (F495L and N458Y), but none of these changes were statistically significant. Thus, introduction of these PfK13 mutations alone did not alter the sensitivities of these transgenic 3D7 parasites to other antimalarial drugs.

**TABLE 2:** IC_50_ values of 10 different antimalarial drugs for parasites with mutated and WT PfK13

Parasite	IC_50_ (nM)^*a*^									
	AMQ	AS	AM	DHA	CQ	LMF	MFQ	PND	PPQ	QN
3D7	11 ± 5	1.7 ± 0.2	1.80 ± 0.01	0.7 ± 0.4	18.7 ± 0.8	3.2 ± 0.4	16.10 ± 0.04	1.7 ± 0.4	18 ± 2	25 ± 6
WT	13 ± 4	1.5 ± 0.2	1.8 ± 0.1	0.6 ± 0.4	19 ± 2	5 ± 2	15 ± 1	2.0 ± 0.1	19.2 ± 0.3	23.8 ± 0.6
F446I	12 ± 4	1.4 ± 0.1	1.60 ± 0.03	0.8 ± 0.4	17.5 ± 0.1	5 ± 1	14 ± 2	1.8 ± 0.3	18.90 ± 0.04	22 ± 8
C469Y	11 ± 4	1.5 ± 0.1	1.6 ± 0.3	0.6 ± 0.1	16.8 ± 0.4	10 ± 9	18 ± 8	1.90 ± 0.01	19.8 ± 0.5	23 ± 4
F495L	12 ± 4	1.4 ± 0.1	1.6 ± 0.1	0.8 ± 0.3	15.40 ± 0.03	4.0 ± 0.2	11.96 ± 0.6	1.88 ± 0.07	17.4 ± 0.8	19 ± 5
C580Y	9 ± 8	1.2 ± 0.5	1.8 ± 0.2	0.8 ± 0.4	17.1 ± 0.3	4.1 ± 0.3	16 ± 3	2.0 ± 0.2	18.1 ± 0.1	17 ± 19
N458Y	12 ± 6	1.3 ± 0.1	1.7 ± 0.1	0.9 ± 0.5	19 ± 1	5.4 ± 0.5	13.7 ± 0.6	1.9 ± 0.1	20 ± 3	30 ± 12

a The IC_50_ values (mean ± SD) of 10 different antimalarial drugs were measured in 72-h proliferation assays, with the final parasitemia being determined from the fluorescence intensities measured on a FLUOstar Optima microplate fluorometer using SYBR green staining. Results from three independent experiments performed in duplicate are presented for 3D7 and parasites with WT and mutated (F446I, C469Y, F495L, C580Y, and N458Y) PfK13. AMQ, amodiaquine dihydrochloride dihydrate; AS, artesunate; AM, artemether; DHA, dihydroartemisinin; CQ, chloroquine diphosphate; LMF, lumefantrine; MFQ, mefloquine hydrochloride; PND, pyronaridine; PPQ, piperaquine; QN, quinine.

### *In vitro* fitness of transgenic parasites.

**FIG 3:**
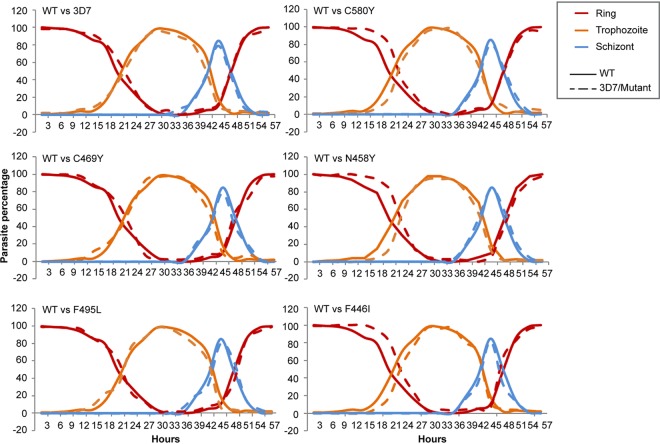
Comparison of intraerythrocytic development patterns between parasites with the introduced WT and mutant *PfK13* genes. Purified schizonts from tightly synchronized parasite lines were used to obtain early-ring-stage parasites. The cultures were then maintained in drug-free medium after sorbitol treatment. Blood smears were made every 3 h, and the percentage of each stage was counted (*y* axis). Ring, trophozoite, and schizont stages are distinguished by different colors, while WT and 3D7 transfected parasites or mutant transfected parasites are shown as continuous and dashed lines, respectively.

**FIG 4:**
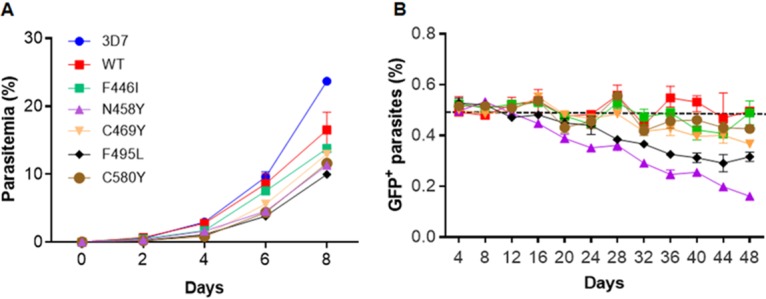
*In vitro* growth of parasites with the WT or mutant PfK13 gene. (A) *In vitro* growth curves of 3D7 and parasites with introduced PfK13 genes (WT and five mutations). The data represent parasitemia counts from three independent experiments performed in duplicate. All parasites were started at 0.1% ring-stage parasitemia, and parasitemia was monitored every other day by using Giemsa-stained smears. (B) Relative growth of the parasite lines carrying the WT and mutant PfK13 genes under *in vitro* competition conditions. All the GFP-tagged WT and mutant PfK13 (F446I, C469Y, F495L, C580Y, and N458Y) parasites were mixed with PTP::WT PfK13 parasites at a 1:1 ratio and cocultured for a period of 48 days in drug-free medium. The cultures were sampled every 3 to 4 days by flow cytometry to determine the proportions of GFP^+^ parasites and total parasites (Deep Red MitoTracker). The *y* axis indicates the average values and standard deviations for the percentage of GFP^+^ cells from two independent assays performed in triplicate. Values of about 50% (dashed line) indicate that the mutated parasites were able to compete well with the PTP::WT PfK13 parasites, whereas numbers <50% indicate a fitness cost.

3D7 parasites with PfK13 mutations did not show a noticeable difference in gross morphology from the WT. To analyze their *in vitro* growth in more detail, we first followed the growth of tightly synchronized parasites at a 3-h interval in a single IDC. Our results showed that parasites with the PfK13 F446I, N458Y, and C580Y mutations had ring-stage development extended for 3 to 4 h. However, subsequent trophozoite development in these parasites was shortened, and they completed schizogony at approximately the same time as the WT PfK13 parasites (Fig. 3). When cultures of all parasite clones were initiated at a 0.1% ring-stage parasitemia, those with the mutant PfK13 alleles grew more slowly than the WT PfK13 parasites (Fig. 4A). The difference became noticeable on day 4 of culture, and on day 8, parasites carrying the N458Y and F495L mutations had significantly lower parasitemias than the WT PfK13 parasites (*P < *0.05, nonparametric Wilcoxon matched-pairs test; Fig. 4A). To determine if the difference in asexual growth of the transgenic parasites also extended to gametocyte development, we measured gametocytemia in the WT and the transgenic 3D7 parasite lines on day 3 and day 10 after the induction of gametocytogenesis. On both days, the gametocytemias were not significantly different among the WT 3D7 and transgenic parasite lines, albeit the WT, C469Y, and N458Y parasites had higher gametocytemias than the other parasite lines tested (Fig. S5).

10.1128/mBio.01134-19.5 FIG S5 Comparison of gametocytemias in WT and mutated PfK13 parasite lines. Daily gametocytemia of all transgenic lines and 3D7 was determined by counting Giemsa-stained gametocytes under a microscope from day 3 through day 10 after induction. Data are shown as the mean ± standard deviation from three replicates at days 3 and 7. None of the values for mutant parasites were significantly different from those for either 3D7 or WT parasites (*P* > 0.1, one-way analysis of variance). Download FIG S5, PPT file, 0.1 MB. Copyright © 2020 Siddiqui et al. 2020 Siddiqui et al. This content is distributed under the terms of the Creative Commons Attribution 4.0 International license.

We next determined the potential fitness cost of the PfK13 mutations in the transgenic 3D7 parasites using an *in vitro* growth competition assay. As a control, we used another parasite line (PTP::PfK13) with N-terminal tagging of PfK13 with a protein C-tobacco etch virus-protein A (PTP) tag (Fig. S1C). The WT PfK13 transfection control did not show any fitness cost, as both PTP::WT PfK13 and GFP::WT PfK13 parasites, when mixed at a 1:1 ratio, maintained an approximately 1:1 ratio during *in vitro* growth for 48 days (Fig. 4B). Values below the dashed line in Fig. 4B, indicating 50% GFP-positive (GFP^+^) parasites, indicate that these parasites grew more slowly and were less fit than the WT PfK13 parasites. All parasites with *PfK13* mutant alleles displayed variable degrees of fitness costs compared to the WT transgenic control and 3D7. Whereas parasite lines with the F446I and C580Y mutations showed only marginal reductions in fitness, those with the N458Y and F495L mutations were significantly less fit (*P < *0.001, nonparametric Wilcoxon matched-pairs test; Fig. 4B). In particular, parasites with the N458Y mutation decreased by 30% during the 48-day growth competition assay.

### PfK13 mutations and cellular stress responses.

**FIG 5:**
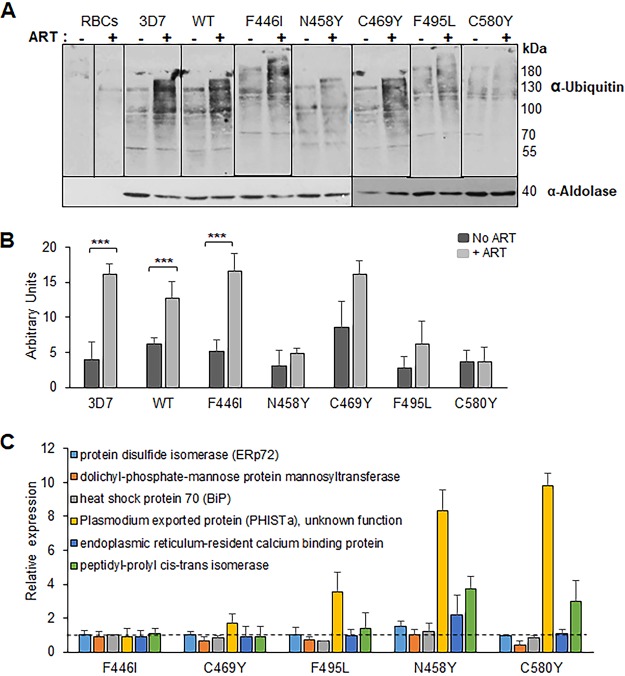
Oxidative stress response in parasites with the WT and mutant *PfK13* genes. (A) Ubiquitination of P. falciparum proteins following ART treatment. Representative Western blots of parasites show protein ubiquitination after ART treatment. Uninfected RBCs or trophozoite-stage parasites of the 3D7, WT, F446I, C469Y, F495L, C580Y, and N458Y strains were treated with 6 μM ART at 3% hematocrit for 90 min at 37°C. Cell extracts were separated by SDS-PAGE and probed with anti-ubiquitin IgG or anti-aldolase antibodies. (B) Quantitation of ubiquitination in parasites after ART treatment. The graph shows the results of a densitometry analysis of the anti-ubiquitin signal normalized to the anti-aldolase signal from three independent experiments (***, *P* <0.005, nonparametric Wilcoxon *t* test; the raw data are presented in Table S2B in the supplemental material). (C) Quantification of relative expression of genes upregulated in ART-resistant field isolates at the early ring stage. RT-quantitative PCR analysis was performed at the early ring stage of WT and isogenic transfected 3D7 parasites carrying the F446I, C469Y, F495L, N458Y, and C580Y mutations for the transcripts of six genes which are upregulated in the ART-resistant parasites (PF3D7_0827900, ERp72; PF3D7_0917900, BiP; PF3D7_1010700, dolichyl-phosphate-mannose protein mannosyl transferase; PF3D7_1372000, plasmodium exported protein [PHISTa]; PF3D7_1108600, endoplasmic reticulum-resident calcium binding protein; and PF3D7_1115600, peptidyl-prolyl *cis-trans* isomerase). Expression levels were normalized to the level for the housekeeping gene seryl-tRNA synthetase, and relative expression (fold change) was calculated using the expression levels in the WT parasites. Values of more than 1 (dashed line) indicate upregulation.

ART treatment is known to result in less accumulation of ubiquitinated proteins in ART-resistant parasites, consistent with engagement of the ubiquitin-proteasome system in ART resistance (34). To determine whether introduction of PfK13 mutations altered the parasites’ stress responses, we measured the levels of protein ubiquitination in WT PfK13 and transgenic parasites at the trophozoite stage in response to a 90-min exposure to DHA. Protein ubiquitination levels in trophozoites of 3D7 and WT PfK13 transfection-control parasites increased after DHA exposure, confirming the involvement of the ubiquitin-proteasome system in response to the ART family of drugs (Fig. 5A and B). Compared with the results for the WT PfK13 control and 3D7, parasites with the N458Y, C580Y, and F495L mutations showed lower levels of accumulation of ubiquitinated proteins after exposure to DHA. However, this level of reduction in protein ubiquitination was not evident in parasites with the F446I and C469Y mutations (Table S2B). To further delve into the mechanism of the stress response in these parasites, we tested the transcript levels of some of the genes which were significantly upregulated in ART-resistant field isolates (37). Compared to the level of expression of *PHISTa* by the transgenic WT control, increased expression of *PHISTa* was shown in all variants except the F446I mutant (Fig. 5C). The N458Y parasites exhibited elevated levels of 4 out of the 6 genes tested, while the C580Y line presented higher levels of transcripts for the peptidyl-prolyl *cis-trans* isomerase and *PHISTa* genes.

## DISCUSSION

In this study, we aimed to genetically determine whether four PfK13 Kelch domain mutations (F446I, N458Y, C469Y, and F495L) present in P. falciparum parasite populations from the China-Myanmar border area confer *in vitro* resistance to ART drugs. We introduced these four mutations into the 3D7 genetic background using the SLI approach, with the recoded PfK13 gene being driven by its endogenous promoter, which allowed us to compare the WT with the isogenic mutants. Though the introduction of all PfK13 mutations into the 3D7 genetic background increased *in vitro* RSA values, only the N458Y mutation conferred ART resistance with a significantly increased *in vitro* RSA value similar to that for the positive transfection control with the C580Y mutation. These findings were further reinforced by the RSA_0-3_ _h_^50%^ assay, which showed a trend similar to that for RSA_0-3_ _h_: all parasites with introduced PfK13 mutations had increased RSA_0-3_ _h_^50%^ values, but the increase was significant only in parasites with the N458Y and C580Y mutations. Further validation of the contribution of these PfK13 mutations to ART resistance came from the reciprocal removal of these mutations from field isolates, which showed that reverting the mutated PfK13 to the WT increased the sensitivity to DHA in these field isolates in the case of all mutations tested except C469Y. Although some of the discrepancies between the findings for field isolates from the China-Myanmar border region where ART resistance has evolved and those for the 3D7 parasite genetic background cannot be reconciled with the current evidence, it is very likely that the genetic backgrounds are a determinant factor. It is noteworthy that the evolution of ART resistance in the GMS is accompanied by several additional mutations in the genome (26) and that the same PfK13 mutations in different genetic backgrounds confer different levels of ART resistance (31).

To elucidate the molecular mechanism underlying PfK13-mediated resistance to ART drugs, we first attempted to identify potential cellular processes in which PfK13 might be involved. PfK13 is consistently expressed during the IDC and localized in distinct cytoplasmic foci (32). The results of a colocalization study were also consistent with a recent report showing the predominant localization of PfK13 to the ER (40). Yet, the discrepancy in the findings of PfK13-PI3P colocalization studies could possibly be due to the use in the previous study of tagged reporter proteins that bind selectively to PI3P to visualize their sites of concentration in live (and fixed) cells, as opposed to the use of commercial anti-PI3P antibodies and conventional IFA in this study with detergents (or methanol) to permeabilize the cells, which may result in the loss of lipids (40). To further investigate the cellular function of PfK13, the PfK13-associated proteome was identified by affinity purification and mass spectrometry (MS) analysis, which revealed a number of proteins potentially involved in the unfolded protein response pathway, protein folding, protein binding, and translation, consistent with the proposed mechanism of PfK13’s involvement in upregulated unfolded protein and oxidative stress responses (37, 40). We also found elevated expression for some of these genes (probably regulating resistance) in our transgenic parasites. Significantly, we detected protein disulfide isomerase (ERp72) from the PfK13 pull-down, and the gene for this enzyme was highly upregulated in the N458Y parasites, hinting at a role in lowering ART sensitivity. ERp72 functions in cell redox homeostasis, protein folding, and the ER stress response (60), but direct evidence of its function in P. falciparum is lacking. Another gene that was upregulated in four of our mutant lines was *PHISTa*. PHIST-family proteins are known to have variable expression in resistant parasites (61) and are central to host cell remodeling, along with PfEMP1 (62). We also tested for eIF2α phosphorylation in our isogeneic parasite lines, as enhanced phosphorylated eIF2α was shown to correlate with high rates of recrudescence following ART (38). We could not detect any differences in the total and phosphorylated forms of eIF2α in these parasites using commercial phosphospecific antibodies for eIF2α (data not shown).

Recent studies demonstrated that a reduced PfK13 abundance in resistant parasites impaired hemoglobin uptake or catabolism (63, 64), but we were unable to detect any significant changes in PfK13 expression or localization after introduction of the PfK13 mutations in the 3D7 strain, as determined by Western blotting, live-cell imaging, and flow cytometry, a finding which is consistent with a few other earlier findings (37, 40). However, by an unknown mechanism, parasites with the PfK13 N458Y and F495L mutations were able to more strongly tolerate the oxidative stress induced by ART drugs, as indicated by lower levels of protein ubiquitination. This result aligns well with earlier findings showing that clinical ART-resistant parasites have an upregulated unfolded protein response pathway (37) and that ART-resistant parasites harboring certain PfK13 mutations have an enhanced stress response involving the ubiquitin/proteasome pathways (34). It is interesting to note that ART-resistant parasites obtained from *in vitro* selection also exhibited genome-wide upregulation of the antioxidant pathways (65).

Ring-stage dormancy and decelerated ring-stage development have been invoked to explain the unique ART resistance phenomenon (36, 66). Since ring-stage parasites are metabolically less active and more able to endure an oxidative onslaught than later stages, it is conceivable that extended ring stages would enable the parasites to better survive ART treatment, which has a short half-life. Population transcriptomics revealed that ART-resistant parasites had decelerated ring-stage development (37). By using highly synchronous ring-stage cultures, we observed a prolonged ring phase in parasites with the F446I, N458Y, and C580Y mutations compared to that in 3D7 and the WT transfectant, even in the absence of drug pressure. Of note, the extended ring stage was not as drastic as that reported in the resistant field isolates, where the ring-stage parasites could persist for up to 30 h, but this phenotype could be more pronounced in the presence of drug, as shown earlier (35, 36).

PfK13 mutations have been shown to incur a fitness cost on the parasites, manifested as lower growth rates as well as reduced fitness in an *in vitro* growth competition assay, as summarized in Table S4 in the supplemental material (57, 67, 68). Different *PfK13* alleles show considerable variations in their impact on parasite fitness, which appear to be governed by the genetic backgrounds of the strains. Straimer et al. found the C580Y mutation to be fitness neutral in recent Cambodian field isolates compared to the effect of the R539T or I543T mutation, whereas the C580Y mutation exerted much higher growth disadvantages in a strain that was culture adapted in 1976, long before ART deployment (57). Two later studies determined that the C580Y mutation carries a greater competitive fitness burden than other PfK13 alleles tested in field isolates obtained from the Thailand-Myanmar border area in 2008 and 2011, respectively (67, 68). Interestingly, the C580Y isolate obtained in 2011 is a clinically resistant parasite strain with a long parasite clearance half-life of 7.84 h (67). In the 3D7 genetic background used in this study, although all mutations introduced brought about various fitness costs, parasites with the C580Y and F446I mutations showed only a marginal reduction in fitness. While this correlated well with the high prevalence of these two mutations in different regions of the GMS (69, 70), the current study also highlights the lack of genetic barriers for the evolution and spread of such mutations in African parasites. It is worrisome that the independent emergence of the C580Y mutation has been detected in South America (71) and Papua New Guinea (72). Furthermore, parasites with all mutations tested in this study appeared to form gametocytes normally, suggesting the potential transmission of these mutant parasites through mosquitoes.

F446I is the most prevalent PfK13 mutation present in northern Myanmar and the China-Myanmar border region (42, 52). This mutation has been associated with *in vivo* delayed parasite clearance (47, 73) and day 3 positive parasitemia (43). Our transgenic 3D7^F446I^ parasites showed RSA values exceeding 1%, a cutoff value used to define ART resistance among field isolates, but these values were not significantly different from the value for the transgenic WT control (19). In a recent study, when the F446I mutation was introduced into the 3D7 and FCC1/HN strains, no statistically significant increase in the RSA value was detected at 700 nM DHA (none of the RSA values were more than 1%), although exposure to lower DHA concentrations did present survival rates significantly higher than those for the WT control (48). In this study, we found an increase in ART sensitivity in one of the field isolates from the China-Myanmar border region after F446I was reverted to the WT, suggesting that the impact of F446I on ART sensitivity varies greatly depending on the genetic backgrounds of the parasites. Moreover, the F446I parasites showed a prolonged ring stage and almost no reduction in fitness, which may contribute to the high prevalence of this mutation in western GMS.

Introduction of the N458Y mutation into 3D7 conferred high RSA values, and reciprocal removal of this mutation in one of the field isolates from the China-Myanmar border region increased the parasite’s sensitivity to DHA. However, the high-level resistance conferred by the N458Y mutation may come with a compromised fitness cost, leading to its low prevalence in areas where malaria is endemic, as observed for the R539T and I543T mutations (57). The N458Y mutation was extremely rare in the population in the China-Myanmar border region, with only 1 isolate with this mutation being identified among 191 clinical isolates (52), and this parasite had the highest RSA value of ∼60% among the field isolates examined in our earlier study (43). The N458Y mutation was also present at a low frequency in the Thai-Myanmar border region, and it was significantly associated with a prolonged parasite clearance half-life of >5 h, day 3 parasite positivity, and an RSA value of >1% in field isolates (49, 50, 74). Our results provide solid genetic evidence supporting these field and laboratory observations associating the N458Y mutation with ART resistance as well as potential reasons for the low prevalence of this mutation in the GMS.

The C469Y mutation was reported at a very low frequency in the China-Myanmar border area and was recently reported from Uganda (1, 11, 43, 52, 54). Only one isolate carrying this mutation from the China-Myanmar border area was found to have day 3 positivity and had an ∼20% *in vitro* ring-stage survival rate (43), but its introduction into 3D7 did not confer a significant increase in ring-stage survival rates. Conversely, the reciprocal removal of C469Y from this field isolate did not significantly alter the parasite’s sensitivity to DHA. Data from the RSA_0-3 h_^50%^, the growth phenotype, and the proteasome stress response to drug treatment further suggest that C469Y is not associated with ART resistance in different genetic backgrounds.

The F495L mutation has been reported at low frequencies from parasite populations in the China-Myanmar border area, Mayotte, and the Democratic Republic of Congo (43, 47, 55, 56), suggesting that it is not associated with ART resistance. Neither clinical nor laboratory studies established any association of F495L with the parasite clearance half-life or *in vitro* phenotype. Although this mutation is near the Y493H mutation, which was described to be associated with delayed parasite clearance (29), it did not produce significantly increased ring survival when introduced into the 3D7 strain. At the same time, the fitness of parasites of the 3D7 background with the F495L mutation was also substantially compromised.

In conclusion, we analyzed the impact of four PfK13 mutations found in northern Myanmar and the China-Myanmar border area on ART resistance using transgenics in 3D7 and mutation reversion in field isolates (Table S5). Our results demonstrated that the introduction of N458Y and C580Y into the 3D7 background significantly elevated the RSA values of the transgenic parasites, whereas other mutations only insignificantly increased the RSA values of the transfectants. In mutation revertants of field isolates, revertants with all mutations except C469Y showed significant decreases in RSA values, highlighting the important role of the genetic backgrounds of the parasites in mediating ART resistance. Compared to C580Y parasites, N458Y parasites showed similar phenotypes in terms of RSA and RSA_0-3 h_^50%^, higher parasite clearance half-lives, a longer ring stage, an elevated proteasomal stress response, and the upregulation of ART resistance-related genes (42, 43, 49, 50), but the mutation had a considerable fitness cost, partially explaining the low prevalence of this mutation in the GMS. While F495L and F446I are potentially involved in ART resistance (Table S5), they alone may not be enough to induce a strong ART resistance phenotype. Generation of these mutations in the African 3D7 background suggests that these mutations may potentially evolve in Africa, emphasizing the importance of heightened surveillance in areas of P. falciparum hyperendemicity.

## MATERIALS AND METHODS

### Parasite culture.

Asexual blood-stage parasites were maintained in O^+^ human red blood cells (RBCs) and a humidified 5% CO_2_ incubator at 37°C as previously described (75). Briefly, parasites were grown in RPMI 1640 with 25 mM NaHCO_3_, 11 mM glucose, 25 mM HEPES (pH 7.4), 0.367 mM hypoxanthine, and 5 μg/liter gentamicin supplemented with 0.5% AlbuMAX II lipid-rich bovine serum albumin (Thermo Fisher Scientific, MA). Synchronization was performed at the ring stage by 5% d-sorbitol treatment (76).

### Plasmid construction and transfection.

pSLI-N-sandwich-loxP (K13) was a gift from Tobias Spielmann. The pSLI-N-sandwich-loxP (PfK13) plasmid contains a recoded *PfK13* gene with its N terminus tagged with the green fluorescent protein (GFP) sandwiched between two FK506-binding protein (FKBP) sequences (2×FKBP::GFP-2×FKBP::K13) (32). To introduce the point mutations (F446I, N458Y, C469Y, F495L, and C580Y) into the recoded *PfK13* gene in this plasmid, site-directed mutagenesis was performed using a Q5 site-directed mutagenesis kit (New England Biolabs, MA) with the primers listed in Table S1A in the supplemental material. To tag the *PfK13* gene with a protein C-tobacco etch virus-protein A (PTP) tag (77), the GFP gene in the pSLI-N-sandwich-loxP (K13) plasmid was replaced with the PTP-coding sequence with the primers listed in Table S1A using an In-Fusion HD cloning kit (TaKaRa Bio USA, Inc., CA). Transfection of the 3D7 parasite and *in vitro* selection were performed as described previously (32, 78, 79). Successful integration was obtained using WR99210 (which selects for the human dihydrofolate reductase [hDHFR]) and DSM1 (which is a dihydroorotate dehydrogenase inhibitor) drug selection. GFP-positive cells were sorted on a Beckman Coulter MoFlo Astrios (Brea, CA) system with 488-nm laser excitation. Correct editing of the *PfK13* gene in the genetically manipulated parasites was verified by PCR, sequencing, and Western blotting.

10.1128/mBio.01134-19.6 TABLE S1 (A) Primers used for site-directed mutagenesis to introduce the point mutations and to replace GFP with PTP. Lowercase letters indicate the mutations introduced. (B) Primers used for RT-PCR. FP, forward primer; RP, reverse primer; BiP, PF3D7_0917900; DPMPM (dolichyl-phosphate-mannose protein mannosyl transferase), PF3D7_1010700; ERCaBP (endoplasmic reticulum-resident calcium binding protein), PF3D7_1108600; PDI (protein disulfide isomerase; ERp72), PF3D7_0827900; PHISTa (plasmodium exported protein), PF3D7_1372000; PPCTI (peptidyl-prolyl *cis-trans* isomerase), PF3D7_1115600. Download Table S1, DOCX file, 0.02 MB. Copyright © 2020 Siddiqui et al. 2020 Siddiqui et al. This content is distributed under the terms of the Creative Commons Attribution 4.0 International license.

10.1128/mBio.01134-19.7 TABLE S2 (A) Raw data for ring-stage survival assay. Percent survival was calculated as (DHA/NE) × 100, where DHA is the number of viable parasites in DHA-exposed wells, and NE is the number of viable parasites in the nonexposed/DMSO-treated well. (B) Raw values from densitometry analysis of Western blots probed with antiubiquitin antibodies for the protein ubiquitination experiment. Data from 3 independent experiments are provided. Download Table S2, DOCX file, 0.01 MB. Copyright © 2020 Siddiqui et al. 2020 Siddiqui et al. This content is distributed under the terms of the Creative Commons Attribution 4.0 International license.

10.1128/mBio.01134-19.8 TABLE S3 IC_50_ values obtained in an RSA_0–3_ _h_^50%^ assay showing the percentage of early-ring-stage parasites (0 to 3 h postinvasion) surviving a 4-h pulse of DHA ranging from 0.6 to 700 nM. *, the IC_50_ values for parasite lines carrying the PfK13 N458Y and C580Y mutations were significantly different from those for 3D7 and WT PfK13 transfection-control parasites (*P* < 0.05, nonparametric Wilcoxon *t* test). Download Table S3, DOCX file, 0.01 MB. Copyright © 2020 Siddiqui et al. 2020 Siddiqui et al. This content is distributed under the terms of the Creative Commons Attribution 4.0 International license.

10.1128/mBio.01134-19.9 TABLE S4 Effect of different K13 mutations on parasite fitness. Download Table S4, XLSX file, 0.01 MB. Copyright © 2020 Siddiqui et al. 2020 Siddiqui et al. This content is distributed under the terms of the Creative Commons Attribution 4.0 International license.

10.1128/mBio.01134-19.10 TABLE S5 Summary of the P. falciparum phenotypes observed with different PfK13 mutations. Download Table S5, DOCX file, 0.1 MB. Copyright © 2020 Siddiqui et al. 2020 Siddiqui et al. This content is distributed under the terms of the Creative Commons Attribution 4.0 International license.

### Western blotting.

Western blotting was performed to detect native or GFP-tagged PfK13 protein expression in the WT 3D7 and GFP::PfK13 transgenic lines. Equal amounts of proteins from different stages were separated on a 10% SDS-PAGE gel, transferred to a nitrocellulose membrane, and probed with monoclonal anti-GFP antibodies (Roche, IN), followed by detection with anti-rabbit immunoglobulin horseradish peroxidase (HRP)-conjugated antibody (Sigma-Aldrich, MO) at 1:5,000. The detected proteins were visualized using an enhanced chemiluminescence (ECL) kit (Invitrogen, MA). Rabbit anti-*Plasmodium* aldolase antibodies (catalog number ab207494; Abcam, Cambridge, UK) were used as a loading control at a 1:3,000 dilution. We also generated antibodies against three different peptides of the K13 protein (which were custom generated using keyhole limpet hemocyanin-conjugated peptides), but only the antibodies generated against amino acids (aa) 239 to 257 showed high titers and detected a single band in Western blots. The PfK13 peptide antibodies were used at a 1:1,000 dilution.

### Indirect immunofluorescence assay (IFA).

For PfK13 localization, 3D7 and the GFP-tagged transgenic parasites at different asexual and sexual stages were fixed using methanol and probed with either rabbit anti-PfK13 antibodies (1:300) or monoclonal anti-GFP antibodies (Roche, IN), followed by Alexa Fluor 488-conjugated goat anti-rabbit IgG antibodies. Parasite nuclei were stained with 4′,6-diamidino-2-phenylindole (DAPI), and the slides were mounted with an antifade reagent (catalog number H-1500; VectaLabs, Australia). For colocalization experiments, 3D7 or GFP::PfK13 parasites were fixed and probed with anti-BiP mouse antisera as the endoplasmic reticulum (ER) marker, anti-HAP mouse sera as the food vacuole marker (catalog number MRA-811A; BEI Resources, VA), or anti-PI3P mouse antibodies (Echelon Biosciences Inc., UT) at a 1:200 dilution (80). Images were acquired on an Olympus FluoView FV1000 epifluorescence microscope. At least 20 images were captured for each colocalization experiment, and Pearson’s correlation coefficients were calculated.

### Protein IP.

Protein immunoprecipitation (IP) from the GFP::K13 parasite line was performed using a GFP-Trap-A kit (Chromotek, Germany) according to the manufacturer’s instructions. Briefly, GFP::PfK13 or 3D7 (control) parasites were harvested after saponin lysis. The pellets were resuspended in a cold lysis buffer (30 mM Tris, pH 7.5, 150 mM NaCl, 0.5 mM EDTA, 0.5% NP-40, 1 mM phenylmethylsulfonyl fluoride [PMSF], Roche protease inhibitors) and incubated on ice for 5 min. Then, the parasites were manually lysed by 60 to 100 strokes in a Dounce homogenizer with a tight pestle and centrifuged at 16,000 × *g* for 20 min at 4°C. The supernatant was incubated with GFP-Trap beads for 4 h or overnight at 4°C, and the beads were washed 3 times with the lysis buffer (without NP-40). The bound proteins were eluted using the elution buffer from the Pierce co-IP kit (Thermo Fisher Scientific). IP eluates were prepared for mass spectrometry (MS)-based proteomics using filter-aided sample preparation. Briefly, proteins were alkylated with iodoacetamide, buffer exchanged with urea followed by ammonium bicarbonate, and finally, digested with trypsin/Lys-C overnight at 37°C. The peptides were eluted and subsequently desalted using C_18_ SPE cartridges (Waters, MA) with a vacuum manifold. Desalted peptides were dried in a vacuum concentrator. The peptides were resuspended in 0.1% formic acid for liquid chromatography-tandem MS (LC-MS/MS) analysis.

Peptides were separated using a 75-μm by 50-cm C_18_ reversed-phase high-performance liquid chromatography column on an Ultimate 3000 ultra-high-performance liquid chromatograph (Thermo Fisher Scientific) with a 120-min gradient (2 to 32% acetonitrile with 0.1% formic acid) and analyzed on a hybrid quadrupole-Orbitrap instrument (Q Exactive Plus; Thermo Fisher Scientific). Full MS survey scans were acquired at a resolution of 70,000. The top 10 most abundant ions were selected for MS/MS analysis.

Raw data files were processed in MaxQuant software (www.maxquant.org) and searched against the UniprotKB P. falciparum protein sequence database. Search parameters included constant modification of cysteine by carbamidomethylation and the variable modification, as well as methionine oxidation. Proteins were identified using the filtering criteria of a 1% protein and peptide false discovery rate (FDR) and at least two unique peptides. Gene Ontology (GO) term enrichment of select gene groups or clusters was carried out using the analysis tools at http://www.PlasmoDB.org.

### Real-time RT-PCR.

For reverse transcriptase (RT) PCR analysis, cDNA was synthesized from 1 μg of total RNA using SuperScript III RT (Invitrogen) and an oligo(dT)_12–17_ primer, and the reaction mixture was diluted to 100 μl. Real-time RT-PCR was performed using a SYBR green PCR kit (Roche, IN) with 1 μl of the cDNA and the primers listed in Table S1B. The relative expression levels of the selected genes at the ring stage were determined using the 2^−ΔΔ^*^CT^* method, with the seryl-tRNA synthetase (STS) gene (*PF07_0073*) being used as the internal reference. All expression values were further normalized with the respective values in the transgenic WT parasites. Data analysis was performed and the threshold cycle (*C_T_*) value was determined as described previously (81).

### Ring-stage survival assays (RSA_0–3_ _h_ and RSA_0–3_ _h_^50%^).

RSA was performed as previously described (19, 27, 43, 57). Briefly, schizonts were purified from tightly synchronized cultures over a gradient of 75% Percoll (Sigma-Aldrich), washed once in RPMI 1640 incomplete medium, and allowed to rupture and invade fresh RBCs for 3 h. The cultures were synchronized again using sorbitol to select for early rings and to eliminate the remaining schizonts. For RSA_0–3 h_, ring-stage parasites (0 to 3 h postinvasion) at 1% parasitemia and 1% hematocrit were exposed to 700 nM DHA for 6 h, followed by a single wash. After culture for 66 h, ∼10,000 RBCs were blind counted on thin blood smears to count viable parasites. The RSA_0–3_ _h_^50%^ value was also evaluated for these parasites and was defined as the DHA concentration required to kill 50% of the parasites when ring-stage parasites (0 to 3 h postinvasion) are exposed to increasing DHA concentrations (0.6 to 700 nM). After 4 h of incubation, the cells were washed four times, followed by two transfers of cells to new 96-well plates as described previously (57, 58). Parasite survival was then assessed by counting 100,000 total cells by flow cytometry on a Beckman Coulter MoFlo Astrios flow cytometer using the GFP signal and MitoTracker Deep Red dye staining (Invitrogen). For all assays, parallel dimethyl sulfoxide (DMSO)-treated controls (0.1%) were used, and survival rates were expressed as the ratios of viable parasites in DHA-exposed and DMSO-exposed samples. RSA_0–3_ _h_^50%^ values were calculated by nonlinear curve fitting of log-transformed data using the GraphPad Prism (v5) program (La Jolla, CA).

### *In vitro* drug sensitivity assays.

The *in vitro* susceptibilities of the WT and mutant parasites to 10 antimalarial drugs, amodiaquine dihydrochloride dihydrate (AQ), artemether (AM), chloroquine (CQ), piperaquine (PPQ), mefloquine (MFQ), quinine (QN), lumefantrine (LMF), pyronaridine (PND), artesunate (AS), and DHA, were tested using a SYBR green I-based assay as described previously (43, 82, 83). PPQ was purchased from Chongqing Kangle Pharmaceutical Co. (Chongqing, China), CQ, MFQ, QN, and ART drugs were purchased from Sigma-Aldrich, while LMF and PND were from Kunming Pharmaceutical Co. (Kunming, Yunnan, China). The stock solutions were prepared as described before (43). To determine the 50% inhibitory concentration (IC_50_), synchronized ring-stage parasites were cultured with serial dilutions of each drug at 1% hematocrit and 0.5% parasitemia in a 96-well plate. Wells with no drug and only RBCs were used as a positive control and the background, respectively. After 72 h of incubation, the plates were wrapped and frozen at −20°C for at least 16 h. The cultures were then thawed and lysed using 100 μl lysis buffer (20 mM Tris-HCl [pH 7.5], 5 mM EDTA, 0.08% Triton X-100, 0.008% saponin in phosphate-buffered saline [PBS; 137 mM NaCl, 2.7 mM KCl, 10 mM Na_2_HPO_4,_ 1.8 mM KH_2_PO_4_], 0.2 μl SYBR green I) (84). The plates were incubated at 37°C for 1 h in the dark after thorough mixing, and fluorescence intensities were measured using a FLUOstar Optima microplate reader (BMG Labtech Inc, NC) at excitation and emission wavelengths of 485 and 520 nm, respectively. Percent growth was calculated against the positive control after subtracting the RBC background signal. IC_50_s were calculated using the GraphPad Prism (v5) program by constructing a dose-response curve. Each experiment was performed three times independently, each with two technical replicates. 3D7 was included in all experiments as an internal reference strain.

### Phenotype analysis.

To measure parasite propagation, schizont-stage parasites from tightly synchronized cultures were purified using 75% Percoll and allowed to rupture and invade erythrocytes for 2 h. Unruptured schizonts were eliminated using sorbitol treatment. Synchronized ring-stage cultures were cultured in 24-well plates at 0.1% rings and 2% hematocrit. The medium was replenished every 24 h, and parasitemia was examined daily for 8 days using Giemsa-stained smears and flow cytometry. After two cycles, the cultures were maintained at 0.2% hematocrit to sustain high parasitemias. Cell cycle progression was monitored using similarly synchronized cultures with a starting parasitemia of 1%. Giemsa-stained smears were read every 3 h for 60 h (85). Three independent biological repeats were performed. For comparing the levels of gametocytemia in mutant and WT parasites, a modified method of gametocyte induction was followed to obtain highly synchronous gametocyte cultures (86). Gametocytemia was determined by counting the gametocytes in at least 5,000 RBCs on Giemsa-stained thin smears for each parasite line.

### Protein ubiquitination in parasites.

Ubiquitinated proteins were analyzed as described previously (34). We used the less potent parent drug ART for treatment, as it was shown to produce a more pronounced response in ubiquitination (34). Briefly, 5% trophozoite parasites from WT and PfK13 mutant strains were treated with 6 μM ART for 90 min at 37°C, followed by washing with PBS containing 20 mM *N*-ethylmaleimide, 2 mM PMSF, an antiprotease mixture, 0.5 mM EDTA, and the cOmplete Mini EDTA-free protease inhibitor mixture (Roche). The RBC membrane was lysed by saponin treatment. Parasite pellets were lysed and separated on 4 to 12% gradient SDS-PAGE gels. Western blotting was performed using polyclonal rabbit antiubiquitin antibodies (1:500 dilution; catalog number 631634; EMD Millipore Corp., MA), followed by goat anti-rabbit immunoglobulin HRP-conjugated antibodies (Sigma-Aldrich) at 1:5,000. The blots were visualized using an enhanced chemiluminescence (ECL) kit (Invitrogen). Rabbit anti-*Plasmodium* aldolase antibodies were used as loading controls at 1:3,000. Densitometry analysis was performed for each lane of the gel using ImageJ software, and all data were normalized according to the aldolase signal.

### *In vitro* growth competition assay.

To determine the potential fitness cost associated with the PfK13 mutations, we performed a mixed-culture competition assay using 7 different parasite lines: PTP::WT, GFP::WT, GFP::F446I, GFP::N458Y, GFP::C469Y, GFP::F495L, and GFP::C580Y. All GFP-tagged parasites (WT or mutant) were mixed with the PTP::WT parasites in a 1:1 ratio at a 3% ring-stage parasitemia. One-fourth of the parasites were used for flow cytometry every 4 days. Cultures were reduced to half and replenished with fresh blood at 50% hematocrit. The ratio of GFP^+^ parasites to the total parasitemia measured by 100 nM MitoTracker Deep Red staining was determined using flow cytometry for an average of 48 days. Experiments were conducted independently three times in duplicate, and the percentage of GFP^+^ parasites was plotted over time. Each time, a total of 50,000 events were read per well.

### Statistical analysis.

Statistical analysis was performed using the GraphPad Prism (v5) program. The geometric mean of the IC_50_ and the 95% confidence interval (CI) were calculated by fitting the drug response data to a sigmoid curve. A nonparametric Wilcoxon matched-pairs test or one-way analysis of variance was used to compare the mean values between treatment groups. Differences were considered significant at a *P* value of <0.05.
