# Supplementary figures and images for: Role of Plasmodium falciparum Kelch 13 Protein Mutations in P. falciparum Populations from Northeastern Myanmar in Mediating Artemisinin Resistance
Source: mBio. 2020 Feb 25;11(1):e01134-19. doi: 10.1128/mBio.01134-19 (PMC7042691; doi:10.1128/mBio.01134-19)

## Slide 1
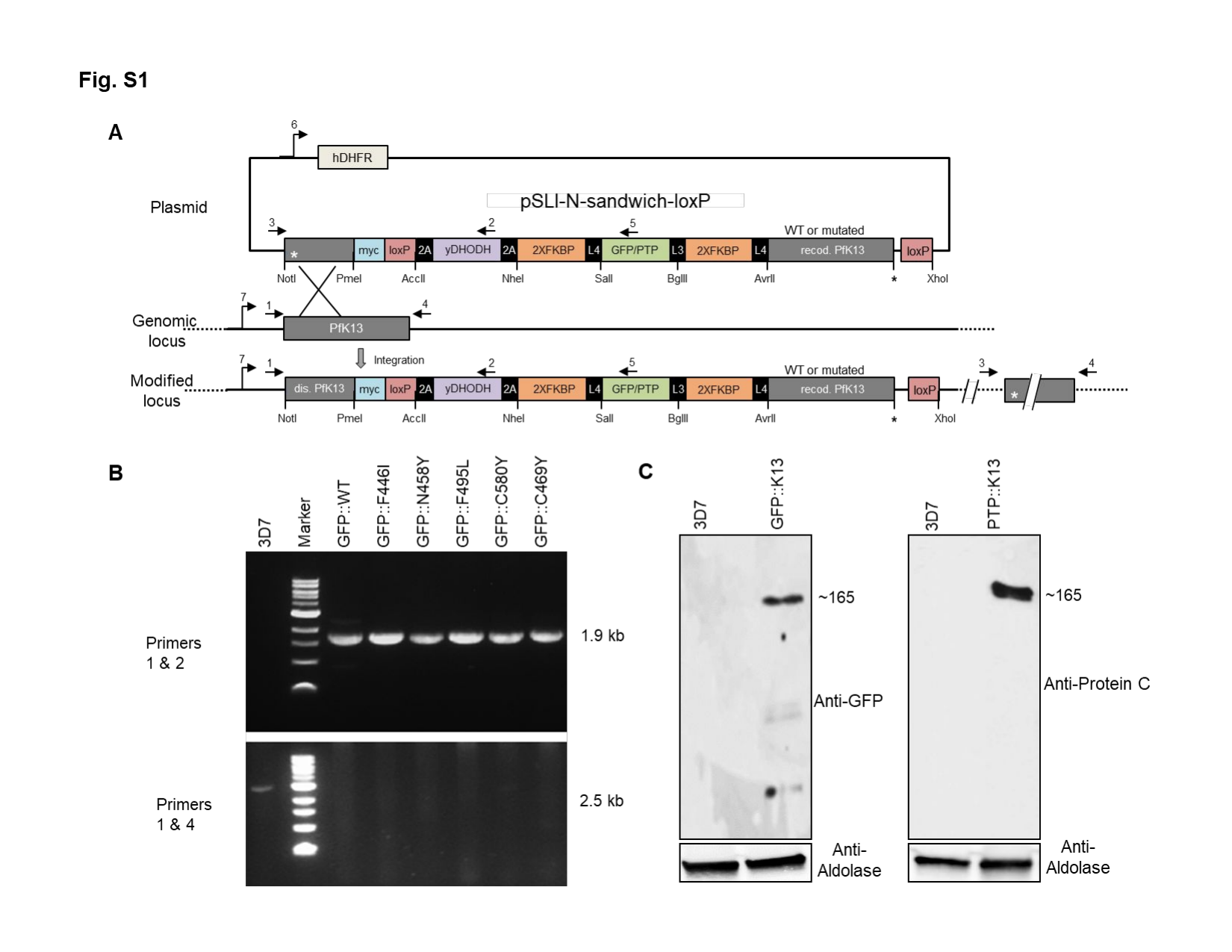

## Slide 2
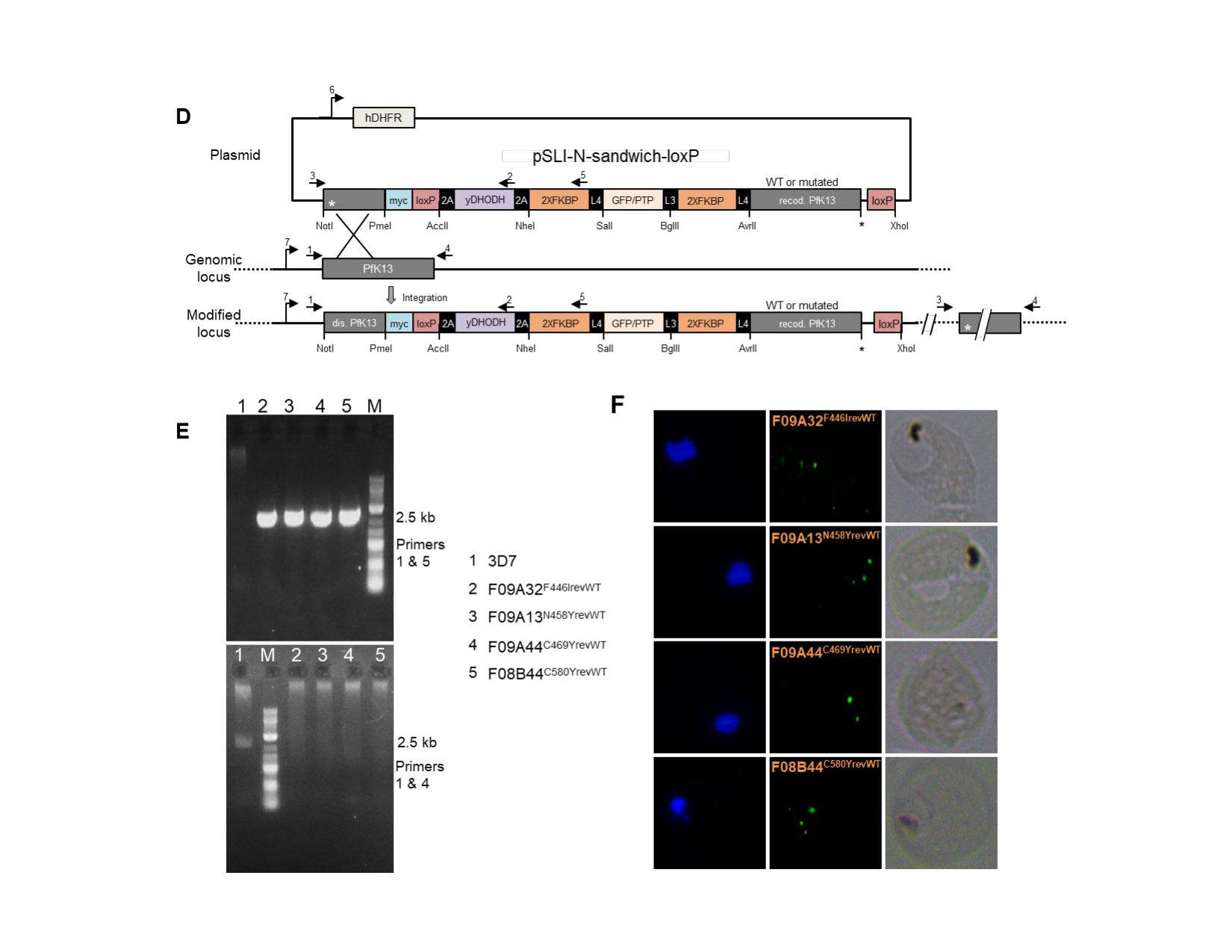

Supplement: FIG S1 [file mBio.01134-19-sf001.ppt]

## Slide 1
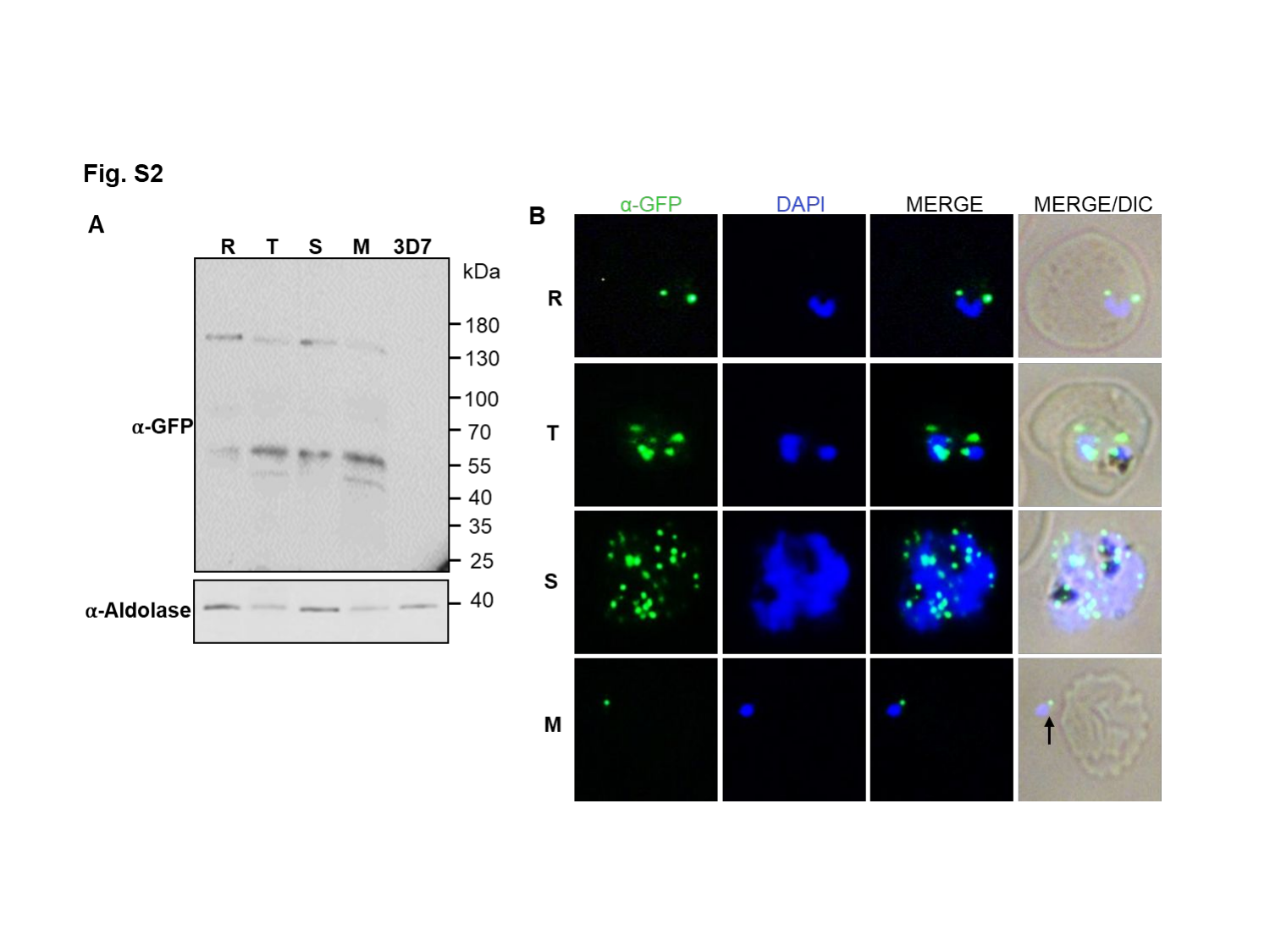

## Slide 2
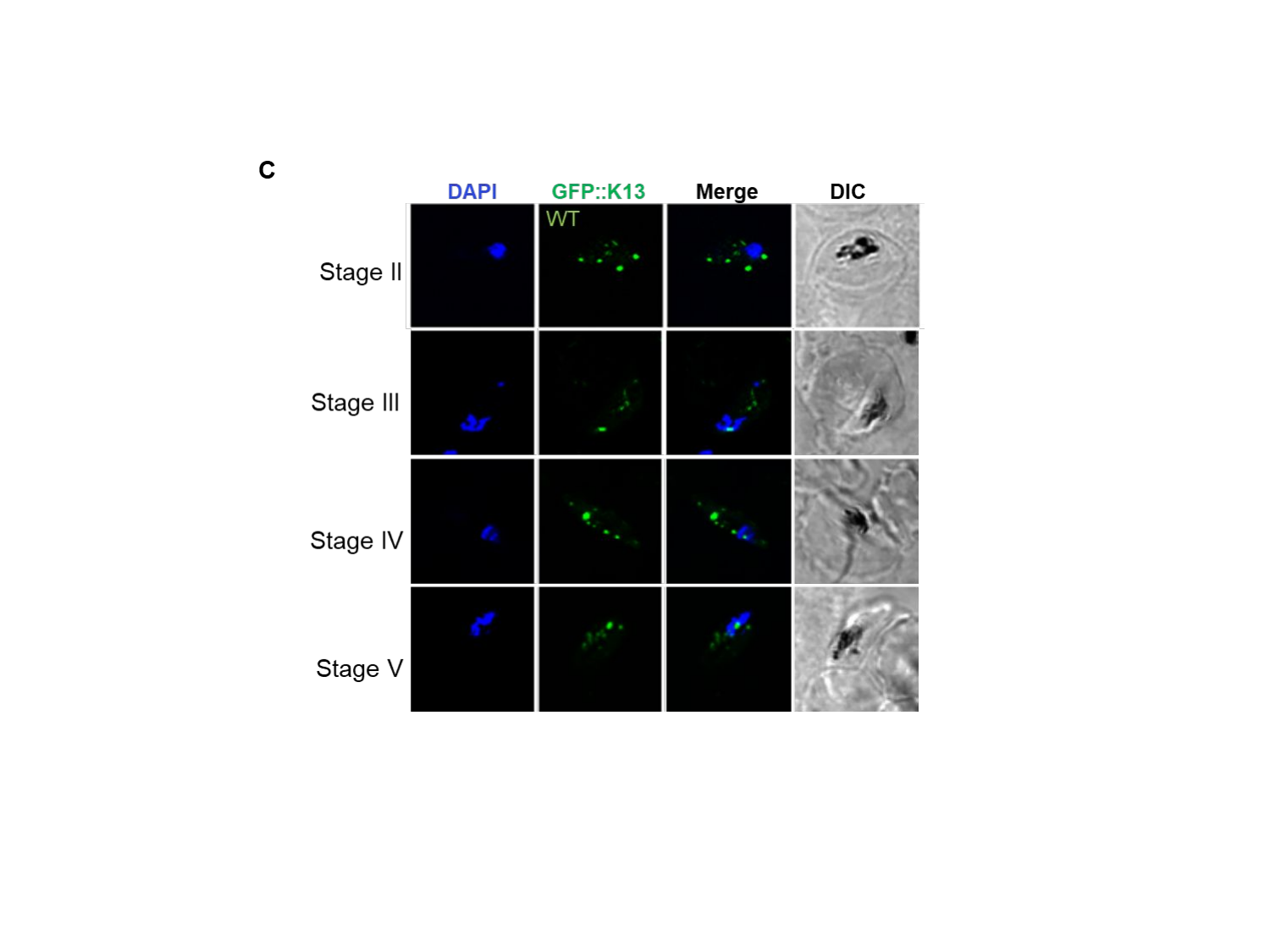

Supplement: FIG S2 [file mBio.01134-19-sf002.ppt]

## Slide 1
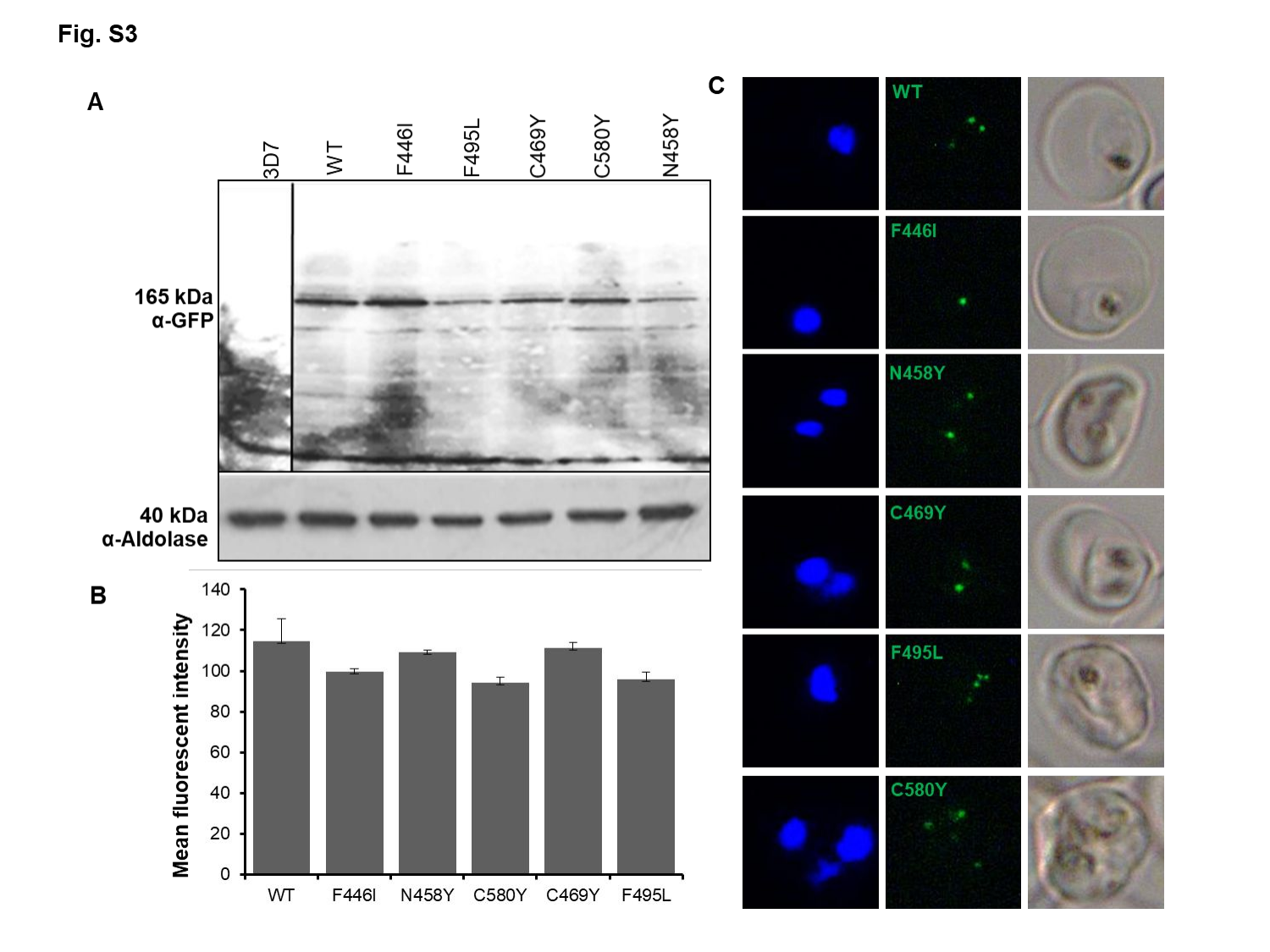

## Slide 2
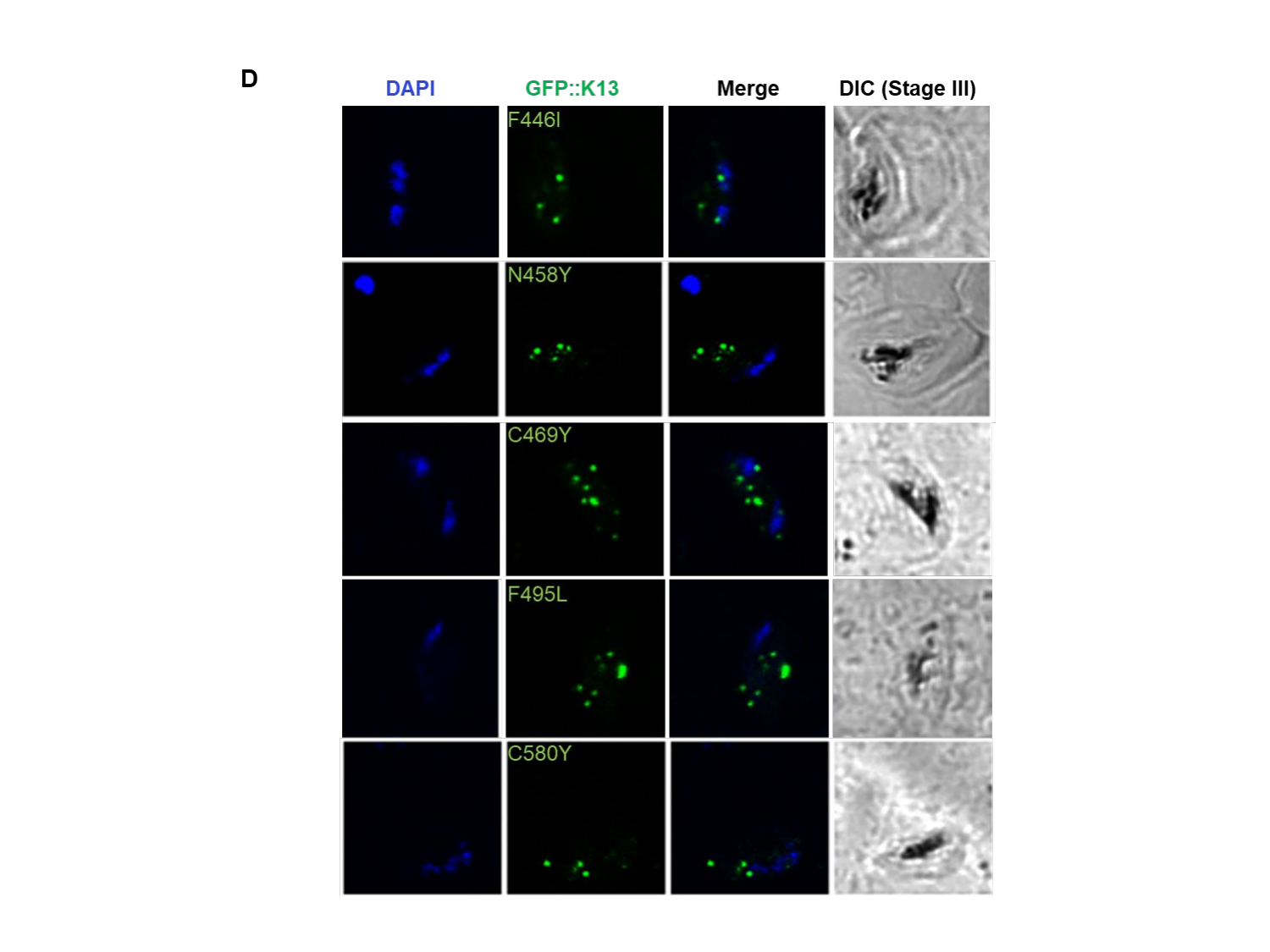

Supplement: FIG S3 [file mBio.01134-19-sf003.ppt]

## Slide 1
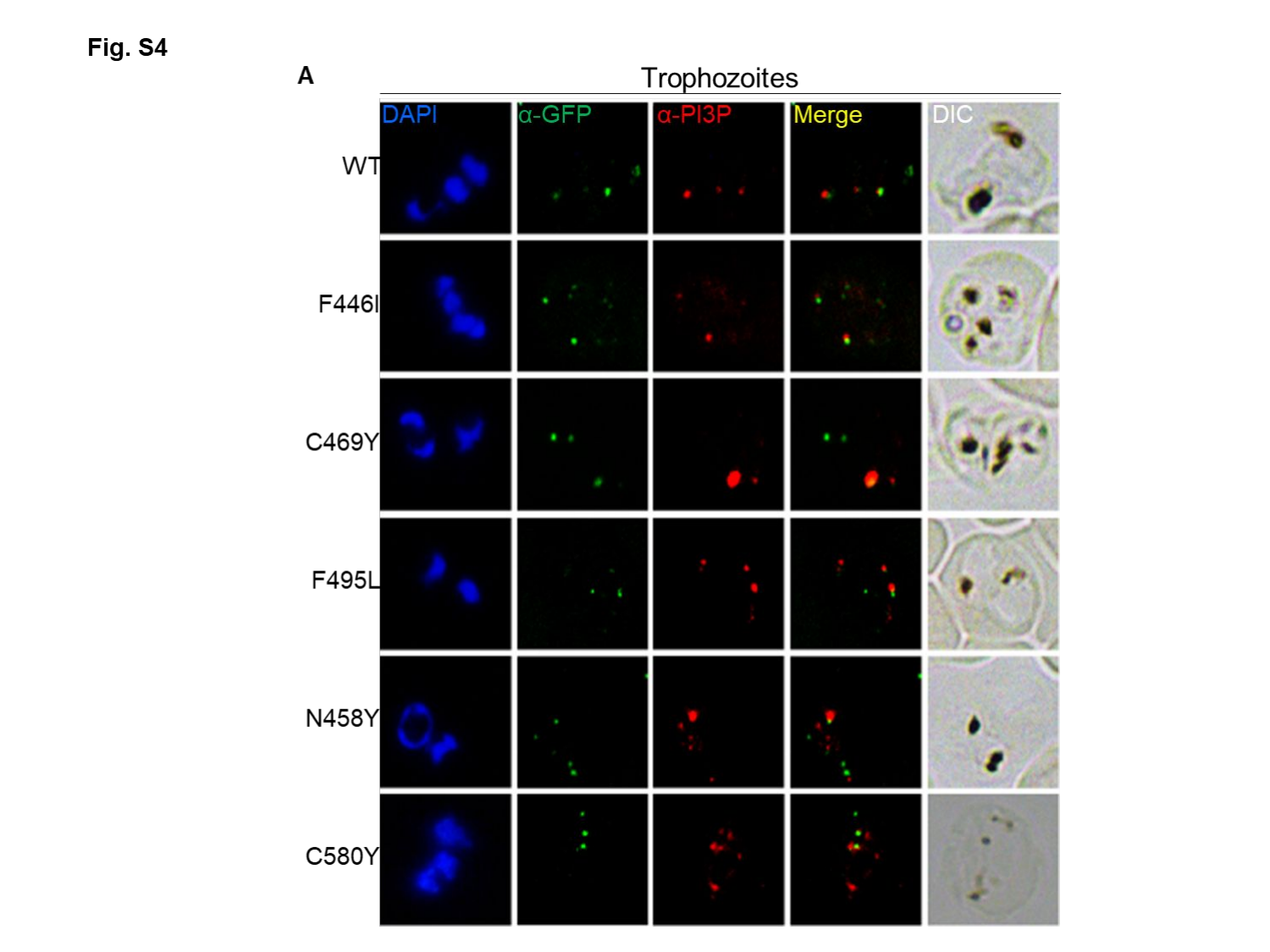

## Slide 2
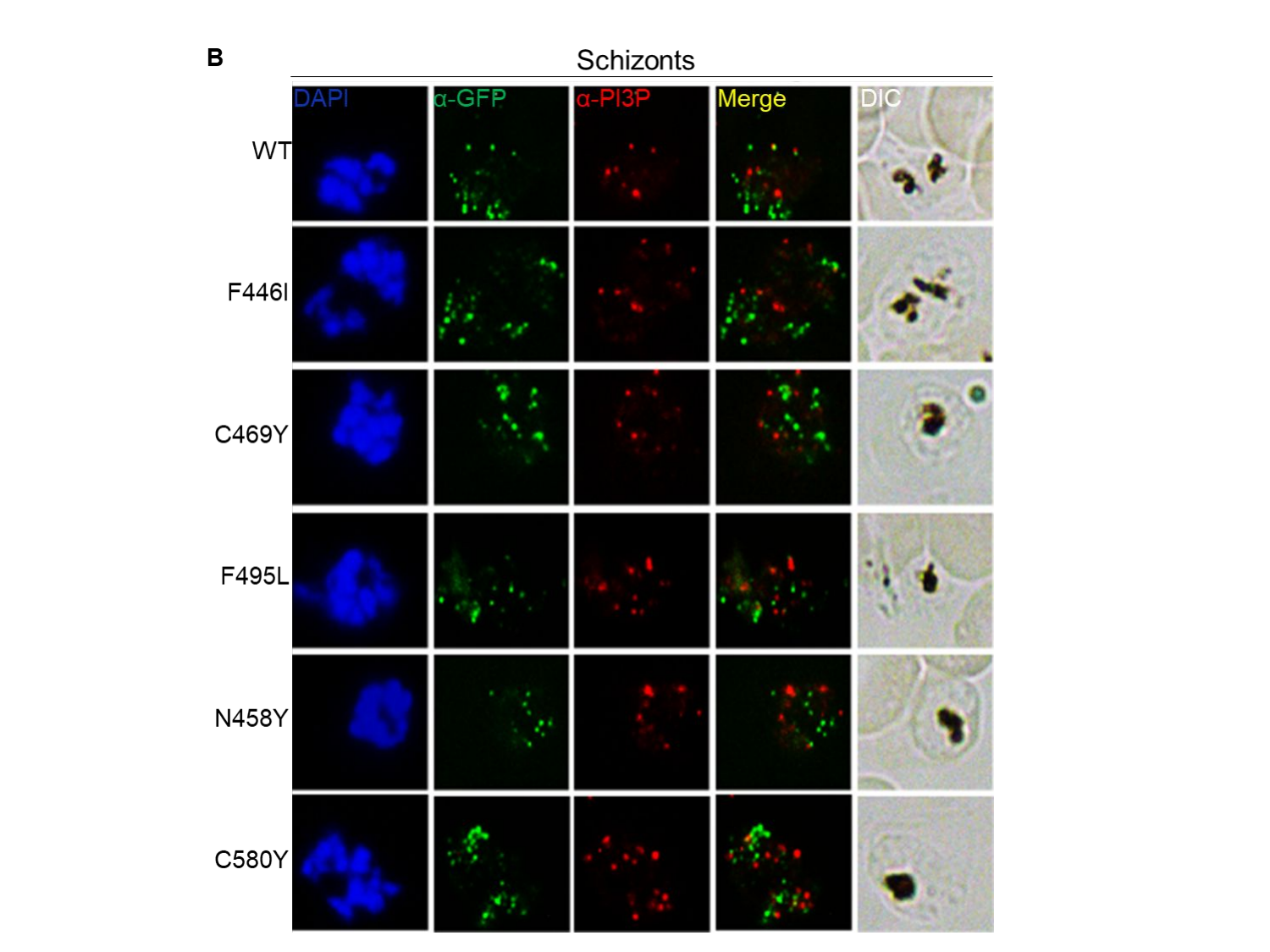

Supplement: FIG S4 [file mBio.01134-19-sf004.ppt]

## Slide 1
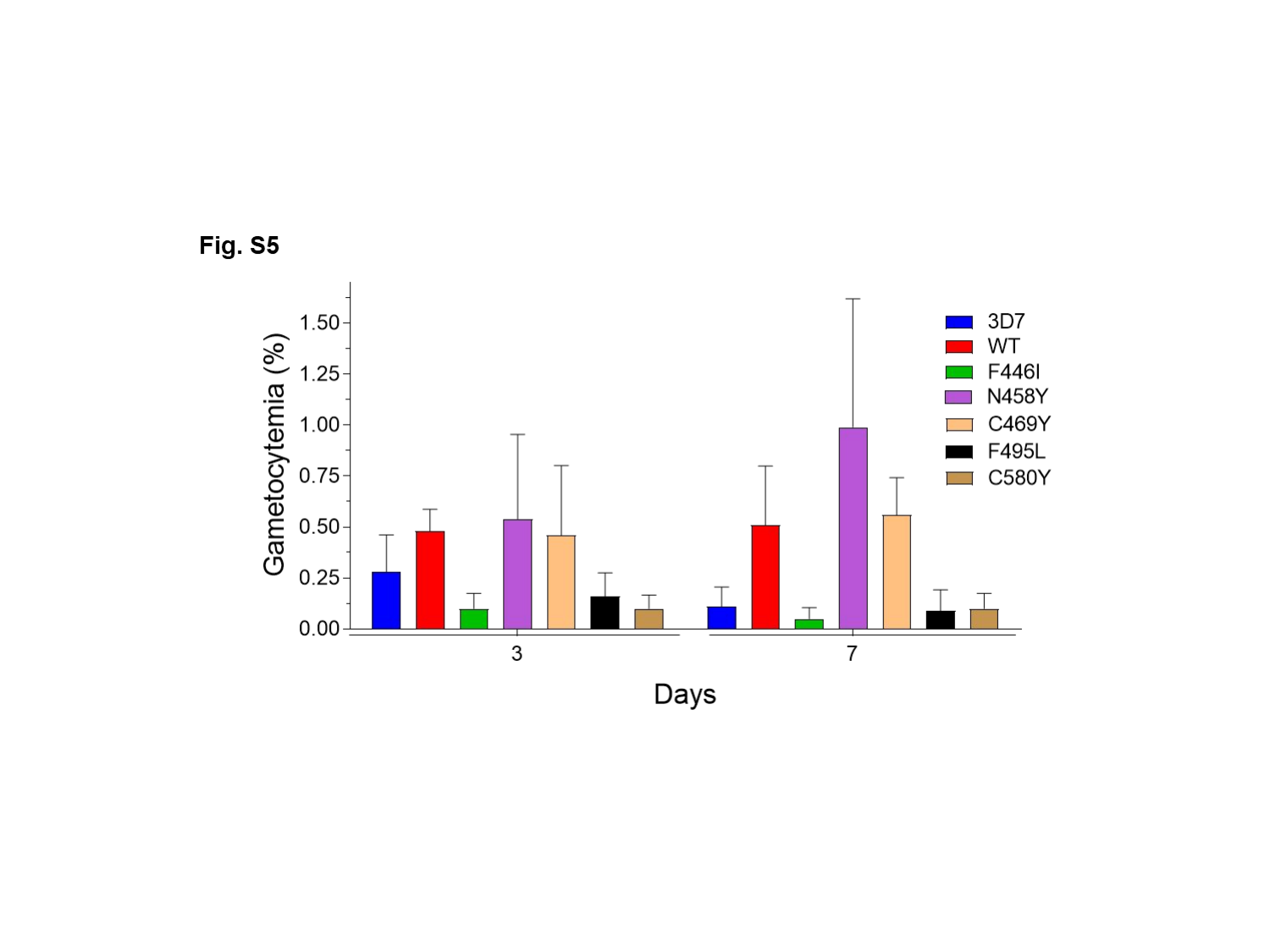

Supplement: FIG S5 [file mBio.01134-19-sf005.ppt]
