# Supplementary material for: Role of Plasmodium falciparum Kelch 13 Protein Mutations in P. falciparum Populations from Northeastern Myanmar in Mediating Artemisinin Resistance
Source: mBio. 2020 Feb 25;11(1):e01134-19. doi: 10.1128/mBio.01134-19 (PMC7042691; doi:10.1128/mBio.01134-19)
Supplement: TABLE S1 [file mBio.01134-19-st001.docx]

**Table S1A.** Primers used for site-directed mutagenesis to introduce the point mutations and to replace GFP with PTP. Lower case letters indicate the mutations introduced.

| **Primers** | **Sequence (5’-3’)** |
| --- | --- |
| FC580YSLI | AGTGCAATGTaCGTAGCATTC |
| RC580YSLI | AGACCTTGGAGTGTTTAATGGA |
| FF446ISLI | TCCTTTGGTTaTCTGCATTGGA |
| RF446ISLI | AAAGGCAAGAACTTTATTCCGTA |
| FN458YSLI | TGAGTACTTGtACAGTATGGAG |
| RN458YSLI | ACTCCGTCGAAACCTCCAATG |
| FC469YSLI | TCACAGCAGTaTTGGCGAATG |
| RC469YSLI | TATGTCCAACAACTCCATACTG |
| FF495LSLI | TTTTGTATGTAcTCGGAGGAAAC |
| RF495LSLI | AGTTGTTTAAAACTGCAGAACCG |
| PTP_cloningSLI_infusion F | GCAGGAAGGgtcgacatgCTGGAAGATCAGGTCGATCC |
| PTP_cloningSLI_infusion R | CAGCAGCAGCAgatctGGTTGACTTCCCCGCGGAA |

**Table S1B.** Primers used for RT PCR. FP: Forward Primer, RP: Reverse Primer, BiP: PF3D7_0917900, DPMPM (dolichyl-phosphate-mannose protein mannosyl transferase): PF3D7_1010700, ERCaBP (endoplasmic reticulum-resident calcium binding protein): PF3D7_1108600, PDI (protein disulfide isomerase, ERp72): PF3D7_0827900, PHISTa (Plasmodium exported protein): PF3D7_1372000, PPCTI (peptidyl-prolyl cis-trans isomerase): PF3D7_1115600.

| **Primers** | **Sequence 5'-3'** |
| --- | --- |
| BiP FP | CGAAATTGAAGATATAGTTG |
| BiP RP | TTTCATATTTAGCATCATCC |
| DPMPM FP | AACTACTAATAAGAATGAGG |
| DPMPM RP | CCTATTAAATACCCATTCG |
| ERCBP FP | GATCCTGCTCATGAAAGT |
| ERCBP RP | CATCACCATAATCAGTTAAT |
| PDI FP | TGGAGGCGGGAGAACA |
| PDI RP | CATTATCTTCTGAAGTATATT |
| PHISTa FP | GACAAGGAAAGGGTGAC |
| PHISTa RP | CATAACTATTTAATTGTCTTG |
| PPCTI FP | CATAGTAAAAGAGGATTATTA |
| PPCTI RP | TATCTAACCCTTCAACTAC |
