# Supplementary material for: Role of Plasmodium falciparum Kelch 13 Protein Mutations in P. falciparum Populations from Northeastern Myanmar in Mediating Artemisinin Resistance
Source: mBio. 2020 Feb 25;11(1):e01134-19. doi: 10.1128/mBio.01134-19 (PMC7042691; doi:10.1128/mBio.01134-19)
Supplement: TABLE S2 [file mBio.01134-19-st002.docx]

**Table S2A.** Raw data for ring stage survival assay. Percentage survival (%) = (DHA/NE) x 100.

DHA: viable parasites in DHA exposed wells. NE: viable parasites in the non-exposed/DMSO well.

| **Isolates** | **RSA1** | **RSA2** | **RSA3** | **Mean** | **SD** |
| --- | --- | --- | --- | --- | --- |
| **3D7** | 0.13 | 1 | 0 | 0.375 | 0.5 |
| **3D7^WT^** | 2.84 | 1.89 | 0.99 | 1.9 | 0.9 |
| **3D7^F446I^** | 8.62 | 1.92 | 3.89 | 4.8 | 3.4 |
| **3D7^N458Y^** | 24.57 | 24.54 | 29.65 | 26 | 3 |
| **3D7^C469Y^** | 3.00 | 3.49 | 4.03 | 3.5 | 0.5 |
| **3D7^F495L^** | 8.64 | 3.22 | 6.88 | 6 | 3 |
| **3D7^C580Y^** | 46.73 | 19.82 | 25.30 | 31 | 14 |
| **F09A32^F446I^** | 5.46 | 8.21 | 8.18 | 7.3 | 1.6 |
| **F09A13^N458Y^** | 45.76 | 42.02 | 37.86 | 42 | 4 |
| **F09A44^C469Y^** | 10.42 | 5.58 | 11.93 | 9.3 | 3.3 |
| **F08B44^C580Y^** | 9.76 | 12.21 | 8.39 | 10 | 2 |
| **F09A32^F446IrevWT^** | 3.56 | 2.89 | 2.75 | 3.1 | 0.4 |
| **F09A13^N458YrevWT^** | 2.99 | 1.91 | 1.74 | 2.2 | 0.7 |
| **F09A44^C469YrevWT^** | 7.82 | 6.75 | 8.73 | 7.8 | 1.0 |
| **F08B44^C580YrevWT^** | 0 | 0 | 1 | 0.3 | 0.6 |

|  | Exp 1 | | Exp 2 | | Exp 3 | |
| --- | --- | --- | --- | --- | --- | --- |
| ART | - | + | - | + | - | + |
| 3D7 | 5.3 | 17.7 | 1 | 16 | 5.6 | 15 |
| WT | 5.3 | 15.2 | 6.98 | 9.9 | 6.1 | 13.1 |
| F446I | 3.99 | 17.5 | 6.9 | 13.5 | 5.2 | 18.1 |
| N458Y | 1.6 | 4.2 | 2.7 | 5.9 | 5.6 | 4.6 |
| C469Y | 4.5 | 14.2 | 12.1 | 15.9 | 9.1 | 18 |
| F495L | 3.4 | 2.6 | 3.9 | 9.8 | 1.1 | 6 |
| C580Y | 5.5 | 6.1 | 3.1 | 2.8 | 2.6 | 2.1 |

**Table S2B.** Raw values from densitometry analysis of western blots probed with anti-ubiquitin antibodies for total protein ubiquitination, normalized with signal for aldolase in each sample. Data from 3 independent experiments is provided.
