# Supplementary material for: Role of Plasmodium falciparum Kelch 13 Protein Mutations in P. falciparum Populations from Northeastern Myanmar in Mediating Artemisinin Resistance
Source: mBio. 2020 Feb 25;11(1):e01134-19. doi: 10.1128/mBio.01134-19 (PMC7042691; doi:10.1128/mBio.01134-19)
Supplement: TABLE S3 [file mBio.01134-19-st003.docx]

**Table S3**. IC_50_ values obtained in an RSA_0-3 h_^50%^ assay showing the percentage of early ring-stage parasites (0 to 3 h post invasion) surviving a 4-h pulse of DHA ranging from 0.6 to 700 nM.

| Parasite strains | IC_50_ (nM) |
| --- | --- |
| 3D7 | 4 **±** 2 |
| WT | 5 **±** 3 |
| F446I | 7 **±** 3 |
| C580Y | 16 **±** 3* |
| C469Y | 6 **±** 3 |
| F495L | 8 **±** 2 |
| N458Y | 12 **±** 3* |

*IC_50_ values of parasite lines carrying the PfK13 N458Y and C580Y mutations are significantly different from those of 3D7 and WT PfK13 transfection control parasites (P < 0.05, Nonparametric Wilcoxon t-test).
