# Supplementary material for: Role of Plasmodium falciparum Kelch 13 Protein Mutations in P. falciparum Populations from Northeastern Myanmar in Mediating Artemisinin Resistance
Source: mBio. 2020 Feb 25;11(1):e01134-19. doi: 10.1128/mBio.01134-19 (PMC7042691; doi:10.1128/mBio.01134-19)
Supplement: TABLE S5 [file mBio.01134-19-st005.docx]

**Table S5.** Summary of the *P. falciparum* phenotypes observed with different PfK13 mutations.

|  | **F446I**  **^(^**[**^1-4^**](#_ENREF_1)**^)^** | **N458Y**  **^(^**[**^3^**](#_ENREF_3)**^,^** [**^5-7^**](#_ENREF_5)**^)^** | **C469Y**  **^(^**[**^1^**](#_ENREF_1)**^,^** [**^3^**](#_ENREF_3)**^,^** [**^4^**](#_ENREF_4)**^,^** [**^8-10^**](#_ENREF_8)**^)^** | **F495L**  **^(^**[**^1^**](#_ENREF_1)**^,^** [**^3^**](#_ENREF_3)**^,^** [**^4^**](#_ENREF_4)**^,^** [**^8^**](#_ENREF_8)**^,^** [**^10-12^**](#_ENREF_10)**^)^** | **C580Y**  **^(^**[**^13-17^**](#_ENREF_13)**^)^** |
| --- | --- | --- | --- | --- | --- |
| RSA reported^#^ | Yes | Yes | Yes | No | Yes |
| Parasite t1/2^#^ | Higher | High >5h | Not consistent^^^ | Not reported**^$^** | High >5h |
| RSA^*^ | NS | High | NS | NS | High |
| Growth phenotype^*^ | NS slower | Slower | NS slower | Slower | Slower |
| Fitness results^*^ | NS | Lower | Lower | Lower | NS |
| Ring stage^*^ | Longer | Longer | NS | NS | Longer |
| RSA_0-3h_^50% *^ | NS | Higher | NS | NS | Higher |
| Protein Ubiquitination after ART treatment^*^ | Higher | NS | Higher but NS | NS | NS |

**#** From previous studies

* Results from this study

**^** (1 isolate from CM border with elevated t1/2 ([1](#_ENREF_1)), 1 isolate in Uganda but no elevated RSA ([9](#_ENREF_9)), one day-3 positive parasite isolate from CM border ([3](#_ENREF_3)), 2 isolates each from CM border with no data on RSA ([4](#_ENREF_4), [10](#_ENREF_10)).

**$** (1 isolate from CM border but no data on RSA or t1/2 ([1](#_ENREF_1)), 2 isolates from CM border ([10](#_ENREF_10)), 1 isolate from Myotte ([11](#_ENREF_11)), 1 isolate from DR Congo ([12](#_ENREF_12)).

**NS:** Not significant

**CM:** China Myanmar

1. Huang F, Takala-Harrison S, Jacob CG, Liu H, Sun X, Yang H, Nyunt MM, Adams M, Zhou S, Xia Z, Ringwald P, Bustos MD, Tang L, Plowe CV. 2015. A Single Mutation in K13 Predominates in Southern China and Is Associated With Delayed Clearance of Plasmodium falciparum Following Artemisinin Treatment. J Infect Dis 212:1629-35.

2. Wang J, Huang Y, Zhao Y, Ye R, Zhang D, Pan W. 2018. Introduction of F446I mutation in the K13 propeller gene leads to increased ring survival rates in Plasmodium falciparum isolates. Malar J 17:248.

3. Wang Z, Wang Y, Cabrera M, Zhang Y, Gupta B, Wu Y, Kemirembe K, Hu Y, Liang X, Brashear A, Shrestha S, Li X, Miao J, Sun X, Yang Z, Cui L. 2015. Artemisinin resistance at the China-Myanmar border and association with mutations in the K13 propeller gene. Antimicrob Agents Chemother 59:6952-9.

4. Ye R, Hu D, Zhang Y, Huang Y, Sun X, Wang J, Chen X, Zhou H, Zhang D, Mungthin M, Pan W. 2016. Distinctive origin of artemisinin-resistant Plasmodium falciparum on the China-Myanmar border. Sci Rep 6:20100.

5. Boulle M, Witkowski B, Duru V, Sriprawat K, Nair SK, McDew-White M, Anderson TJ, Phyo AP, Menard D, Nosten F. 2016. Artemisinin-Resistant Plasmodium falciparum K13 Mutant Alleles, Thailand-Myanmar Border. Emerg Infect Dis 22:1503-5.

6. Talundzic E, Okoth SA, Congpuong K, Plucinski MM, Morton L, Goldman IF, Kachur PS, Wongsrichanalai C, Satimai W, Barnwell JW, Udhayakumar V. 2015. Selection and spread of artemisinin-resistant alleles in Thailand prior to the global artemisinin resistance containment campaign. PLoS Pathog 11:e1004789.

7. Tun KM, Imwong M, Lwin KM, Win AA, Hlaing TM, Hlaing T, Lin K, Kyaw MP, Plewes K, Faiz MA, Dhorda M, Cheah PY, Pukrittayakamee S, Ashley EA, Anderson TJ, Nair S, McDew-White M, Flegg JA, Grist EP, Guerin P, Maude RJ, Smithuis F, Dondorp AM, Day NP, Nosten F, White NJ, Woodrow CJ. 2015. Spread of artemisinin-resistant Plasmodium falciparum in Myanmar: a cross-sectional survey of the K13 molecular marker. Lancet Infect Dis 15:415-21.

8. Fairhurst RM, Dondorp AM. 2016. Artemisinin-Resistant Plasmodium falciparum Malaria. Microbiol Spectr 4.

9. Ikeda M, Kaneko M, Tachibana SI, Balikagala B, Sakurai-Yatsushiro M, Yatsushiro S, Takahashi N, Yamauchi M, Sekihara M, Hashimoto M, Katuro OT, Olia A, Obwoya PS, Auma MA, Anywar DA, Odongo-Aginya EI, Okello-Onen J, Hirai M, Ohashi J, Palacpac NMQ, Kataoka M, Tsuboi T, Kimura E, Horii T, Mita T. 2018. Artemisinin-Resistant Plasmodium falciparum with High Survival Rates, Uganda, 2014-2016. Emerg Infect Dis 24:718-726.

10. Wang Z, Shrestha S, Li X, Miao J, Yuan L, Cabrera M, Grube C, Yang Z, Cui L. 2015. Prevalence of K13-propeller polymorphisms in Plasmodium falciparum from China-Myanmar border in 2007-2012. Malar J 14:168.

11. Torrentino-Madamet M, Collet L, Lepere JF, Benoit N, Amalvict R, Menard D, Pradines B. 2015. K13-Propeller Polymorphisms in Plasmodium falciparum Isolates from Patients in Mayotte in 2013 and 2014. Antimicrob Agents Chemother 59:7878-81.

12. Mvumbi DM, Bobanga TL, Kayembe JN, Mvumbi GL, Situakibanza HN, Benoit-Vical F, Melin P, De Mol P, Hayette MP. 2017. Molecular surveillance of Plasmodium falciparum resistance to artemisinin-based combination therapies in the Democratic Republic of Congo. PLoS One 12:e0179142.

13. Ariey F, Witkowski B, Amaratunga C, Beghain J, Langlois AC, Khim N, Kim S, Duru V, Bouchier C, Ma L, Lim P, Leang R, Duong S, Sreng S, Suon S, Chuor CM, Bout DM, Menard S, Rogers WO, Genton B, Fandeur T, Miotto O, Ringwald P, Le Bras J, Berry A, Barale JC, Fairhurst RM, Benoit-Vical F, Mercereau-Puijalon O, Menard D. 2014. A molecular marker of artemisinin-resistant Plasmodium falciparum malaria. Nature 505:50-5.

14. Ashley EA, Dhorda M, Fairhurst RM, Amaratunga C, Lim P, Suon S, Sreng S, Anderson JM, Mao S, Sam B, Sopha C, Chuor CM, Nguon C, Sovannaroth S, Pukrittayakamee S, Jittamala P, Chotivanich K, Chutasmit K, Suchatsoonthorn C, Runcharoen R, Hien TT, Thuy-Nhien NT, Thanh NV, Phu NH, Htut Y, Han KT, Aye KH, Mokuolu OA, Olaosebikan RR, Folaranmi OO, Mayxay M, Khanthavong M, Hongvanthong B, Newton PN, Onyamboko MA, Fanello CI, Tshefu AK, Mishra N, Valecha N, Phyo AP, Nosten F, Yi P, Tripura R, Borrmann S, Bashraheil M, Peshu J, Faiz MA, Ghose A, Hossain MA, Samad R, et al. 2014. Spread of artemisinin resistance in Plasmodium falciparum malaria. N Engl J Med 371:411-23.

15. Menard D, Khim N, Beghain J, Adegnika AA, Shafiul-Alam M, Amodu O, Rahim-Awab G, Barnadas C, Berry A, Boum Y, Bustos MD, Cao J, Chen JH, Collet L, Cui L, Thakur GD, Dieye A, Djalle D, Dorkenoo MA, Eboumbou-Moukoko CE, Espino FE, Fandeur T, Ferreira-da-Cruz MF, Fola AA, Fuehrer HP, Hassan AM, Herrera S, Hongvanthong B, Houze S, Ibrahim ML, Jahirul-Karim M, Jiang L, Kano S, Ali-Khan W, Khanthavong M, Kremsner PG, Lacerda M, Leang R, Leelawong M, Li M, Lin K, Mazarati JB, Menard S, Morlais I, Muhindo-Mavoko H, Musset L, Na-Bangchang K, Nambozi M, Niare K, Noedl H, et al. 2016. A Worldwide Map of Plasmodium falciparum K13-Propeller Polymorphisms. N Engl J Med 374:2453-64.

16. Ghorbal M, Gorman M, Macpherson CR, Martins RM, Scherf A, Lopez-Rubio JJ. 2014. Genome editing in the human malaria parasite Plasmodium falciparum using the CRISPR-Cas9 system. Nat Biotechnol 32:819-21.

17. Straimer J, Gnadig NF, Witkowski B, Amaratunga C, Duru V, Ramadani AP, Dacheux M, Khim N, Zhang L, Lam S, Gregory PD, Urnov FD, Mercereau-Puijalon O, Benoit-Vical F, Fairhurst RM, Menard D, Fidock DA. 2015. Drug resistance. K13-propeller mutations confer artemisinin resistance in Plasmodium falciparum clinical isolates. Science 347:428-31.
